# Transcriptional and proteomic insights into phytotoxic activity of interspecific potato hybrids with low glycoalkaloid contents

**DOI:** 10.1186/s12870-021-02825-w

**Published:** 2021-01-22

**Authors:** Katarzyna Szajko, Jarosław Ciekot, Iwona Wasilewicz-Flis, Waldemar Marczewski, Dorota Sołtys-Kalina

**Affiliations:** 1 Plant Breeding and Acclimatization Institute, Młochów Research Centre, Platanowa 19 st, 05-831 Młochów, Poland; 2 Ludwik Hirszfeld Institute of Immunology and Experimental Therapy, Laboratory of Biomedical Chemistry, Rudolfa Weigla 12 st, 53-114 Wrocław, Poland

**Keywords:** Allelopathy, Diploid hybrid, Glucan endo-1,3-beta-glucosidase, Leptine II, Solamargine, *Solanum chacoense*, *Solanum tuberosum*, Solasonine, Threonine deaminase

## Abstract

**Background:**

Glycoalkaloids are bioactive compounds that contribute to the defence response of plants against herbivore attack and during pathogenesis. Solanaceous plants, including cultivated and wild potato species, are sources of steroidal glycoalkaloids. *Solanum* plants differ in the content and composition of glycoalkaloids in organs. In wild and cultivated potato species, more than 50 steroidal glycoalkaloids were recognized. Steroidal glycoalkaloids are recognized as potential allelopathic/phytotoxic compounds that may modify the growth of target plants. There are limited data on the impact of the composition of glycoalkaloids on their phytotoxic potential.

**Results:**

The presence of α-solasonine and α-solamargine in potato leaf extracts corresponded to the high phytotoxic potential of the extracts. Among the differentially expressed genes between potato leaf bulks with high and low phytotoxic potential, the most upregulated transcripts in sample of high phytotoxic potential were anthocyanin 5-aromatic acyltransferase-like and subtilisin-like protease SBT1.7-transcript variant X2. The most downregulated genes were carbonic anhydrase chloroplastic-like and miraculin-like. An analysis of differentially expressed proteins revealed that the most abundant group of proteins were those related to stress and defence, including glucan endo-1,3-beta-glucosidase acidic isoform, whose expression level was 47.96× higher in potato leaf extract with low phytotoxic.

**Conclusions:**

The phytotoxic potential of potato leaf extract possessing low glycoalkaloid content is determined by the specific composition of these compounds in leaf extract, where α-solasonine and α-solamargine may play significant roles. Differentially expressed gene and protein profiles did not correspond to the glycoalkaloid biosynthesis pathway in the expression of phytotoxic potential. We cannot exclude the possibility that the phytotoxic potential is influenced by other compounds that act antagonistically or may diminish the glycoalkaloids effect.

**Supplementary Information:**

The online version contains supplementary material available at 10.1186/s12870-021-02825-w.

## Background

Allelopathy is a broadly understood phenomenon that refers to multidirectional interactions among organisms (plants, bacteria, viruses and fungi) that involve the release of compounds called allelochemicals into the environment [1]. Plant allelopathy between donor and acceptor plants is mainly negative in nature and impairs plant growth, development and/or germination. The ability to synthetize and release allelopathic compounds, especially in plant-plant and plant-pathogen interactions, is an important aspect of allelopathy since it determines plant survival and proper development during biotic stresses [2]. To distinguish allelopathy in ecosystems from research on allelopathic interactions in laboratory, term ‘phytotoxicity’ is used, describing negative interactions between donors and acceptors [1]. A lot of laboratory research focused on recognition of phytotoxic potential use water extracts as phytotoxic factor. Water extracts most closely resemble leaching of compounds from plant organs, that occurs under natural conditions (leaching by rain, dew) [3, 4].

An integral component of allelopathic interactions are allelochemicals that are secondary metabolites derived from three biosynthetic pathways: the shikimate, isoprenoid/mevalonate and polyketide pathways [5]. Based on their origin, secondary metabolites can be divided into three groups: phenylpropanoids, terpenoids, and polyketides. These compounds are distinguished from primary metabolites by their characteristic structure (unique carbon skeleton), which makes them specialized for responses to environmental conditions and biotic stresses. Secondary metabolites are involved in allelopathic interactions but principally in plant defence against pathogens [6].

Solanaceous species have been integral parts of human civilizations as food sources and drugs for thousands of years. All of the approximately 180 tuber-bearing *Solanum* species are indigenous to Latin America. They occur in a wide range of environmental conditions from Mexico in the north to Chile in the south and occupy various habitats [7]. The ability of wild potatoes to adapt and acclimatize to environmental conditions makes them a rich source of variability in biotic and abiotic stress resistance [8]. Steroidal glycoalkaloids, nitrogen-containing steroidal glycosides, are secondary metabolites that occur naturally in most plant organs of *Solanum* species [9]. They are biosynthesized by the sterol branch of the mevalonic acid/isoprenoid pathway [10]. There are many types of glycoalkaloids (GAs) in potato germplasm. Fifty-six GAs, including α-solanine, α-solasonine, α-solamargine, α-chaconine, and leptine II, were revealed in tubers of wild species and cultivated potato [11]. The GA content in tubers is markedly lower than that in potato leaves [12].

α-Solanine and α-chaconine possess antibacterial, fungicidal and insecticidal properties [13], can be classified as phytoalexins and are synthesized in response to pathogen infection [14]. Their biological activity depends mostly on their chemical structures, and α-chaconine is more active than α-solanine on the growth of fungi such as *Alternaria Brassicicola, Phytophthora. medicaginis,* and *Rhizoctonia solani* [15]. In our previous paper, we confirmed the potato phytotoxic potential (PP) against the test plant mustard (*Sinapis alba* L.), specie often used as aftercorp [16]. We demonstrated a significant role of total glycoalkaloid (TGA) content in the expression of PP among wild potato species and potato hybrids, and presented that some clones with low TGA content may possess PP.

For better understanding of processes/phenomena that may be directly or indirectly implicated with PP of potato under low TGA content, we used transcriptomic and proteomic approaches. It is known that allelopathic/phytotoxic interactions are determined directly by allelopathic compounds however, their content or profile in plant’s organs is regulated not only at the genetic level but also undergo coordination of metabolic pathways. Transcriptomic and proteomic studies may provide complementary knowledge to integrative metabolite profiling and better understanding of allelopathy/phytotoxicity phenomena at plant system level [17]. In the present study, we showed that potato PP under low TGA content is directly related with GAs composition.

Contrasting F1 individuals differing in PP and GAs from a cross of an interspecific *Solanum* hybrid with *S. chacoense* were used for comprehensive analyses of transcriptomic and proteomic profiles.

## Results

### Evaluation of total glycoalkaloid, glycoalkaloid, total phenolic and total flavonoid contents

Two bulk samples, C and D, that had similar total glycoalkaloid (TGA) contents (2.7 and 2.6 μg ml^− 1^) and various PP were used (Table 1). Both samples had similar concentrations of α-solanine (0.23 and 0.26 μg ml^− 1^) and α-chaconine (0.14 and 0.18 μg ml^− 1^) (Table 1). α-Solasonine was detected in only C at a concentration of 1.69 μg ml^− 1^ and consisted 40.8% of GAs, while the most frequent glycoalkaloid in D was leptine II (66.7%, at a concentration of 1.22 μg ml^− 1^). The total flavonoids (TF) were significantly higher in D, while total phenolics (TP) were at similar levels in both samples (Table 1). The average retention time and mass of GAs found in the samples are presented in Additional File 1, and mass spectra are presented in Additional File 2.

**Table 1:** Concentration, frequency of glycoalkaloids, total phenolics and total flavonoids in potato leaf extract of C and D samples

**Bulks**	**C**		**D**	
**Glycoalkaloids**^**a**^	Frequency [%]	Concentration [μg ml^-1^]	Frequency [%]	Concentration [μg ml^-1^]
α-Solasonine	40.8	1.69±0.06	0.0	0.00
α-Solamargine	39.9	1.65±0.05	9.3	0.17±0.04
α-Solanine	5.6	0.23±0.02	14.2	0.26±0.06
α-Chaconine	3.4	0.14±0.07	9.8	0.18±0.04
Leptine II^b^	10.4	0.43±0.01	66.7	1.22±0.22
	**C**		**D**	
**TGA concentration [μgml**^**-1**^**]**^**c**^	2.7±0.4		2.6±0.6	
**TP concentration**^**c**^	23.7±2.7		29.4±4.7	
**TF concentration**^**c**^	4.2±0.1		4.9^*^±0.2	
**Phytotoxic potential [%]**	40.6±7.2		0.0^d*^±5.8	

^a^ measured using mass spectrometry

^b^ counted as equivalent of α-solanine

^c^ colorimetric measurement (±SD)

^d^ length of PLE-treated plants the same as in control

^e^deviated from bulk C (*t-student test*)

### Profiles of differentially expressed gene and protein

GAs have been recognized as compounds with protective activity against pathogens, pests and herbivores. Recently, we confirmed that GAs present in potato leaf extract (PLE) exhibit PP. PLE of wild species and hybrids inhibited mustard growth, and TGA content was negatively correlated with mustard root and seedling length [16]. Based on this finding, we addressed the question of which factors/phenomena may play a significant role in the expression of PP under low TGA (2.7 μg ml^− 1^) content in PLE? We analysed the PP of potato in relation to gene and protein expression in potato leaves and GA content in PLE.

Leaf RNA of bulk samples C and D was analysed using the BGISEQ-500 (BGI, China) platform. The total number of raw reads ranged from 28,723,117 to 28,804,382. A total of 23,836 differentially expressed transcripts were identified between libraries of C and D. We analysed the most differentially regulated transcripts with false discovery rates (FDR-adjusted *p*-value < 0.05) as a threshold, finding 3125 up- and 3479 downregulated transcripts. The results for all differentially expressed genes (DEGs) after comparison of the D vs. C data are presented in Additional File 3. The top 10 most up- and downregulated transcripts are presented in Table 2. The most upregulated transcripts in C were anthocyanin 5-aromatic acyltransferase-like and subtilisin-like protease SBT1.7, transcript variant X2, with log2 fold changes (FCs) of 9.45 and 9.19, respectively. The most downregulated genes were carbonic anhydrase chloroplastic-like and miraculin-like, with log2 FCs of − 9.94 and − 9.28, respectively.

**Table 2:** The top 10 most abundant transcripts in down-regulated and up-regulated genes from RNA-seq data, after comparison mRNA samples D vs. C

Gene name	Locus	log2 FC^**a**^	FDR ***p***-value^**b**^
**Up-regulated DEGs**			
Anthocyanin 5-aromatic acyltransferase-like	LOC102605147	9.45	9.20E-14
Subtilisin-like protease SBT1.7, transcript variant X2	LOC102596363	9.19	9.51E-13
Putative uncharacterized protein YER190C-A	LOC107057927	8.24	5.49E-10
Probable disease resistance protein At1g61310, transcript variant X1	LOC102600040	6.68	7.34E-06
1-Aminocyclopropane-1-carboxylate oxidase 5-like	LOC102589195	6.67	1.66E-06
Trans-resveratrol di-O-methyltransferase-like	LOC107057698	6.65	1.61E-06
Uncharacterized LOC102590955	LOC102590955	6.58	1.09E-05
Two-component response regulator ARR2	LOC102596771	6.51	1.90E-05
Uncharacterized LOC107058900	LOC107058900	6.48	1.65E-05
Miraculin-like	LOC102589829	6.27	6.09E-05
**Down-regulated DEGs**			
Carbonic anhydrase, chloroplastic-like	LOC102589374	−9.94	3.32E-20
Miraculin-like	LOC107061746	−9.28	3.78E-13
Cannabidiolic acid synthase-like 2	LOC102604287	−9.17	1.02E-12
21 kDa protein-like	LOC102592643	−9.00	2.55E-12
Putative UPF0481 protein At3g02645	LOC102605914	−9.00	3.09E-12
Peroxidase 16	LOC102581043	−8.40	1.77E-10
Protein detoxification 29-like	LOC102581213	−8.39	8.42E-42
Metallothionein-like protein type 2	LOC102589950	−8.32	3.98E-10
Non-specific lipid-transfer protein 1-like	LOC102599380	−8.22	5.72E-10
Threonine dehydratase biosynthetic, chloroplastic-like	LOC102583664	−8.21	5.59E-10

^a^log2 estimated fold change

^b^FDR adjusted *p*-value

**Fig. 1:**
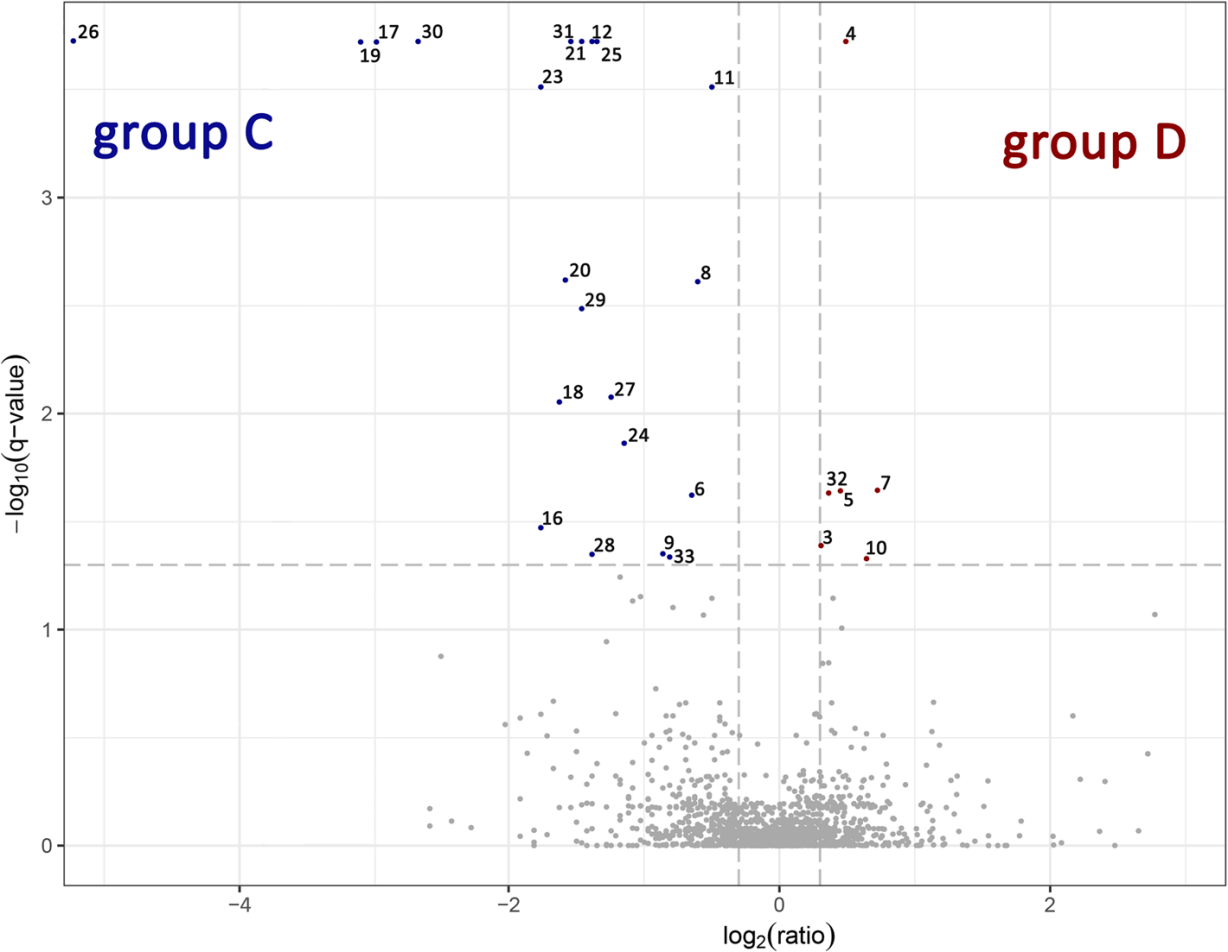
Potato leaf proteins that varied after comparison of bulks D vs. C. The volcano plot shows quantitative abundance of proteins. Only the blue and red dots possess the q-value < 0.05. Volcano plot shows quantitative abundance of proteins. Log-transformed q values (t-test) associated with individual peptides plotted against log-transformed fold change in abundance between the bulk C and D. Proteins that varied qualitatively (no. 1, 2, 13, 14, 15, 22) are not marked on volcano plot. Presented number of varied proteins correspond with protein’s numbers described in Table 2

Liquid chromatography−mass spectrometry analysis of the samples C amd D resulted in 48,836 spectra that were linked to matched peptides, 2125 accessions, and annotated as 3054 proteins. All quantitatively abundant proteins are presented on Fig. 1. Thirty-three differentially expressed proteins (DEPs) were found using the established criteria (q-value< 0.05) (Table 3). The DEPs were divided into five categories according to the system of [18] and assigned based on the UniProt database: primary metabolism, amino acid metabolism, cell structure, protein turnover and stress and defence. The most abundant group of proteins were those related to stress and defence (12 proteins) with glucan endo-1,3-betaglucosidase acidic isoform, whose expression was 47.96× higher in D than in C. Among the DEPs, 3 were characteristic of C: in the category of primary metabolism, there were formamidase and non-symbiotic haemoglobin 2, and in the category of cell structure, there was tetratricopeptide repeat superfamily protein. For D, 3 DEPs were characteristic: 2 in the category cell structure (protein trichome birefringence-like, lysine histidine transporter 1) and 1 in stress and defence (basic endochitinase).

**Table 3:** List of 33 differentially expressed proteins after comparison samples D vs. C

ID	Protein accession numbers^**a**^	Protein	q-value	Peptides^**b**^	Fold change (D/C)^**c**^
**Primary metabolism**					
1	M1CC20 XP_006339814.1	Similar to: formamidase	only in C	14	
2	M1BEV1 XP_006364443.1	Similar to: non-symbiotic hemoglobin 2	only in C	4	
3	M1AL00 XP_006363768.1	Similar to: ferredoxin-dependent glutamate synthase, chloroplastic	0.00015	457	0.81
4	M1CK21, M1CK23 XP_006347295.1	Similar to: chloroplast stem-loop binding protein of 41 kDa b, chloroplastic	0.00028	163	0.71
5	M1AZB4 XP_006349675.1	Similar to: Chloroplast stem-loop binding protein of 41 kDa a, chloroplastic	0.00106	152	0.72
6	P04045 NP_001275215.1	Alpha-1,4 glucan phosphorylase L-1 isozyme, chloroplastic/amyloplastic	0.02116	93	1.50
7	P32811 NP_001275118.1	Alpha-glucan phosphorylase, H isozyme	0.01682	89	0.61
8	M0ZYC1	Protein disulfide-isomerase	0.02141	115	1.45
9	M1AIV9	Pectinesterase	0.00106	94	1.72
**Amino acid metabolism**					
10	M1BCZ5, P54260 NP_001275291.1	Aminomethyltransferase, mitochondrial	0.04385	247	0.78
11	**M1AZP7, P31212 XP_006366786.1**	**Threonine dehydratase biosynthetic (Fragment)**	**0.00162**	**153**	**1.37**
12	M1BH63	Similar to: acetylornithine deacetylase	0.00015	74	2.43
**Cell structure**					
13	M1AJ37, M1AJ38 XP_006351160.1	Similar to: Tetratricopeptide repeat superfamily protein	only in C	3	
14	M1AN84 XP_006362437.1, M1BTR0 XP_006348847.1	Similar to: Protein trichome birefringence-like	only in D	6	
15	M1BT25 XP_006338671.1	Similar to: lysine histidine transporter 1	only in D	5	
16	M1A9U0 XP_006357191.1	Similar to: 30S ribosomal protein S21	0.02471	24	3.28
**Protein turnover**					
17	M1AMY3, M1AMZ0 XP_006353922.1	Similar to: Kunitz-type protease inhibitor D	0.00015	42	7.82
18	P58521, P58518, P58520, M1AKE5, P16348, P17979,	Aspartic protease inhibitor 9 Aspartic protease inhibitor 3 (Fragment) Aspartic protease inhibitor 6 (Fragment) Similar to: Aspartic protease inhibitor 5 Aspartic protease inhibitor 11 Aspartic protease inhibitor 8	0.00161	8	2.94
19	P37842, M1A5P8, M1A5Q3, M1A5P9	Multicystatin, Cysteine proteinase inhibitor	0.00015	87	10.07
20	M1C4F2, M1C4F3 XP_006364268.1	Similar to: Aspartyl protease family protein	0.00131	57	2.87
21	P31427 NP_001305566.1	Leucine aminopeptidase, chloroplastic	0.00015	80	2.58
**Stress and defense**					
22	M1AGK5	Similar to: basic endochitinase	only in D	13	
23	M0ZMG2	Similar to: Acidic endochitinase Q	0.00015	54	3.17
24	M1D578	Peroxidase	0.00132	53	2.11
25	M1APC7, M1APC8, M1APC9 XP_006365633.2	Similar to: Glucan endo-1,3-beta-glucosidase, acidic	0.00015	104	2.42
26	M1APC4	Similar to: Glucan endo-1,3-beta-glucosidase, acidic	0.00015	13	47.96
27	M1CX91	Similar to: glucan endo-1,3-beta-glucosidase, acidic	0.00162	50	2.32
28	M1APC5	Similar to: glucan endo-1,3-beta-glucosidase, acidic	0.00527	16	2.42
29	Q941G6 NP_001275095.1	Pathogenesis-related protein 1b	0.00233	42	2.60
30	M1BPP7 AFW90570.1	Pathogenesis-related protein P2	0.00015	36	5.70
31	M0ZMA9 XP_006340889.1	Similar to: pathogenesis-related protein STH-2	0.00015	80	2.74
32	M1CBM0 XP_006349319.1	Similar to: stromal 70 kDa heat shock-related protein, chloroplastic	0.00713	197	0.77
33	P32111, M0ZQ21, M0ZQ26 XP_006367669.1	Probable glutathione S-transferase	0.04449	63	1.71

^a^ Accession number according to UniProt/NCBI

^b^ Number of peptides matched to predicted protein sequence

^c^ Fold change (D/C) derived by comparison of relative protein intensity between the bulk D and the bulk C, values above fold change > 1 are characteristic for D sample

ns - non significant

bolded – protein that abundance is correlated with its gene expression from RNA-seq experiment

### Functional enrichment analysis

**Fig. 2:**
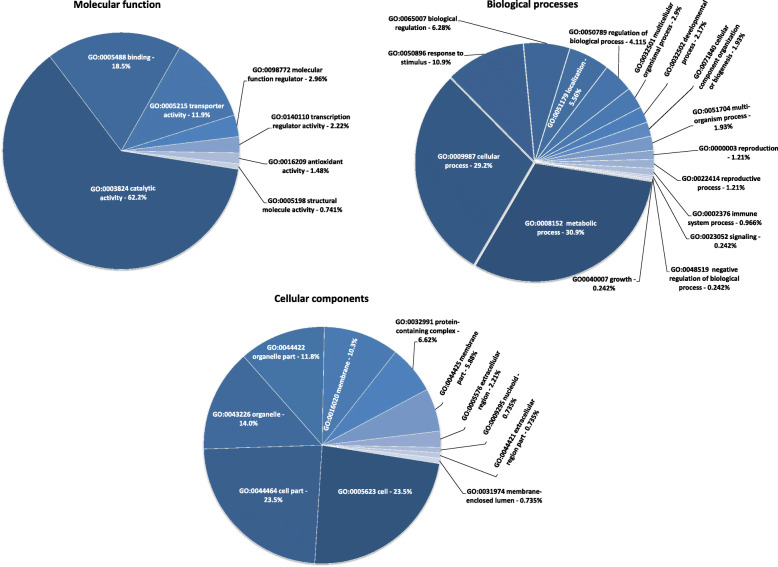
Gene ontology (GO) analysis of potato D vs. C differentially expressed genes. The relative frequencies of GO hits for target genes assigned to the GO functional categories: cellular components, biological processes and molecular functions

Based on the GO analysis, three functional groups were categorized: molecular function (MF), biological process (BP) and cellular component (CC). We identified 34 main GO categories, in three levels (16 GO terms in Biological process, 7 GO terms in Molecular function and 11 GO terms in Cellular component) (Fig. 2). In the MF group, the most significantly enriched GO terms were catalytic activity, binding followed by transporter activity. For the BP group, metabolic process, cellular process and response to stimulus were the GO terms most significantly enriched. With regard to CC, most enriched GO terms were cell, cell part and organelle. All unprocessed GO terms identified from the comparison of D vs. C are shown in Additional File 4.

## Discussion

We previously demonstrate significant involvement of GAs in potato phytotoxic interactions. However, we observed that potato clones with low GAs content also possess phytotoxic abilities. We suspected that PP of clones with low GAs content is determined by various GAs composition. In this purpose we derived a diploid potato population with *S. chacoense* as a paternal donor of PP, to diminish effect of genetic background and selected clones of contrasting PP. *S. chacoense* is a potato wild relative and is known as a source of various steroidal glycoalkaloids, including leptine glycoalkaloids [19]. Depending on the *S. chacoense* accession a lot of glycoalkaloids can be found including leptine I and II, leptinine I and II, α-solanine, α- and β-chaconine, commersonine and demissine; other alkaloids as calystegines and clavepictine, or steroidal saponins as saponin, tuberoside, torvoside or graeculin [20–23]. In the present study, the low TGA content in C and D accompanied various GA patterns among the samples. Most frequently, the GAs α-solasonine and α-solamargine together may play a significant role in PP expression in C. Both compounds were previously recognized in ripe fruits of grey bitter-apple (*Solanum incanum* L.) as an inhibitors of lettuce (*Lactuca sativa* L.) root growth with comparable strength at a concentration of 100 mg dm^− 3^ [24]. Moreover, they may act synergistically when applied jointly in a 1:1 ratio, as was demonstrated on cucumber (*Cucumis sativus* L.) radicle growth when these two compounds were applied separately [25].

The biological activity of GAs was found to be strictly influenced by the configuration and occurrence of sugar moieties [26]. Thus, the composition of GAs may shape the expression and strength of PP. The phytotoxic effect of GAs and their derivatives on cucumber root growth increased with increasing concentration and was dependent on their type [25]. The strongest effect was observed for α-chaconine, which was followed in descending order by α-solanine, α-solamargine, α-solasonine, 6-O-sulfated chaconine and 6-O-sulfated solamargine. To our knowledge, there are no data regarding leptine II allelopathic abilities. The occurrence of foliar leptine glycoalkaloids is directly related to the degree of feeding deterrence of Colorado potato beetle (CPB) [20, 27]. Leptine II has the monoacetylated (C23) aglycone leptinidine. This chemical modification increases leptine toxicity against CPB and human tumour cell lines; however, its phytotoxicity has not yet been evaluated [20, 28].

Glycoalkaloid biosynthesis can be described as a series of actions of glycoalkyloid metabolism (GAME) genes that promote steroidal alkaloid (aglycone) yields [29]. Then, the aglycones undergo glycosylation by different UDP-glycosyltransferases to synthesize various GAs [30]. We found different expression patterns of various UDP-glycosyltransferases (see Additional File 5), especially UDP-glycosyltransferase 90A1 (log2 FC = -3.01), which confirmed different GA landscapes between the samples and may affect PP of PLE. It was recently demonstrated that the pattern of GAs in progeny derived from crosses with *S. chacoense* segregates and may be regulated by 38 genes that are located on chromosome VIII and coexpressed with GAME genes [31]. Comprehensive analysis of DEGs, the GO enrichment, pointing significant participation of metabolic processes (BP-30.9%) and catalytic activity (MF-62.2%) as most differentiating the bulks. This may suggest significant involvement of metabolic pathways in regulation of phytotoxic potential.

We cannot exclude the possibility that PP is influenced by other compounds that act antagonistically or may diminish the GAs effect. As was shown by [15], α-solanine applied together with gallic acid and quercetin diminished the inhibitory effect of α-solanine applied alone on mustard root growth. In the present study, D had a significantly higher TF content than C, which may influence the strength of the phytotoxic effect; however, this difference in concentration was slight.

We suggested that PP expression may be directly related to the qualitative and/or quantitative content of each GA recognized in PLE under low TGA content. Since GAs are involved in plant resistance events and the phytotoxicity of PLE, we assumed that PP of C will be accompanied by increased expression of genes and proteins involved in plant resistance to biotic stress. However, comparison of gene and protein expression profiles in D vs. C did not provide definite evidence to support this concept. Patterns of DEGs involved in the defence response, e.g., endopeptidases, were found, but they were shared between samples. Notably, a gene whose expression is increased in C encodes trans-resveratrol di-O-methyltransferase-like (pterostilbene synthase, EC 2.1.1.240), an enzyme responsible for synthesis of stilbenes, a group of phytoalexins. Most DEGs for this enzyme, which were positioned on different loci, were upregulated in C (see Additional File 3). Transgenic tobacco (*Nicotiana tabacum* L.) lines overexpressing stilbene synthase exhibited increased synthesis of phytoalexins, which was accompanied by resistance to *Botrytis cinerea* infection [32].

The protein profiles of D vs. C do not correspond to gene expression data. The most numerous group of DEPs was involved with stress and defence responses in D. An extremely high fold change was noted for glucan endo-1,3-β-glucosidase acidic isoform (47.96). Plant glucan endo-1,3-β-glucosidases are enzymes that can participate in resistance against pathogens and degradation of cell wall components (β-glucans) during plant development [33, 34]. However, their importance in pathogenesis was debated, since antisense transformants of *N. sylvestris* and tobacco with β-1,3-glucanase deficiency could compensate for the activity of this enzyme by producing a functionally equivalent replacement during infection by tobacco mosaic virus [35].

Protein abundance is controlled by variation at the coding gene itself and by variation mapping to other regions of the genome [36]. Protein synthesis is regulated at many levels, ranging from splicing and mRNA degradation to protein modification to ubiquitination and proteolysis in proteasomes [37]. Therefore, it is not surprising that transcriptome data did not correspond to proteomic profiles (Table 2 and Table 3). We found one DEG, threonine dehydratase biosynthetic (FC = 1.37), whose protein expression is significantly increased in D. Threonine dehydratase, also called threonine deaminase, is an enzyme for isoleucine biosynthesis [38] and acts together with protease inhibitors in the herbivore gut, decreasing dietary proteins and threonine availability. In *Solanum* species, the threonine dehydratase gene has a duplicated paralogue that is coexpressed with genes engaged in herbivore resistance. In lupin (*Lupinus* sp. L.), a high content of threonine was accompanied by an increased level of alkaloids [39]. On the other hand, a higher level of threonine induced the synthesis of alkaloid-pilocarpine in *Pilocarpus vahl* [40], is a good precursor of pyrrolizidine alkaloids in *Senecio douglasii* and participates in the direct biosynthesis of strigosine [41]. One of the proteins characterized for only C was formamidase (EC 3.5.1.49), which is engaged in nitrogen metabolism [42]. The gene encoding this enzyme was also recognized as drought-responsive and regulated by abscisic acid (ABA) [43]. Both enzymes may participate indirectly in GA synthesis due to their involvement in the metabolism of nitrogen, which is inserted into the aglycone skeleton.

## Conclusion

Complex analysis of GAs, together with trancriptomic and proteomic results provided new insight in understanding the expression of phytotoxic abilities of potato. We demonstrated that PP expression under low TGA content may be connected with the GAs composition in PLE. We pointed potential involvement of metabolic pathways (GO enrichment) in direct regulation of GAs biosynthesis and accumulation (genes of GAs main biosynthesis pathway) or indirect regulation by other factors/phenomena, e.g., nitrogen metabolism, and is a result of plant reactions to biotic and abiotic stresses. Comparison of transcriptomic with proteomic revealed gene and protein of threonine dehydratase bisynthetic as common element bonding these two omics and important in expression of potato phytotoxic abilities.

## Methods

### Plant material

Plant material consisted of the potato diploid population 15–1 (F1 progeny, *N* =  166) from a cross of *Solanum* hybrid DG 88–89 (seed parent) and a wild species *S. chacoense* (pollen parent). The maternal clone was generated within the diploid potato breeding program at the Plant Breeding and Acclimatization Institute—National Research Institute, Młochów, Poland. Paternal specie was obtained from National Centre for Plant Genetic Resources, Radzików, Poland (accession POL003:333133). DG 88–89 was the multigenerational hybrid originating from crosses of diploid potato clones. In terms of the genomes, the percentage of *S. tuberosum* in DG 88–89 was 78.3% and that of *S. chacoense* was 15.7%. Maternal clone and paternal species differ with TGA concentration in the PLE and PP. DG 88–89 exhibited a low TGA concentration (5.2 μg ml^− 1^) and nondetectable PP, while *S. chacoense* had a high TGA concentration (55.6 μg ml^− 1^) and high PP (70%) [15]. Three replicates of each of the progeny were grown in a greenhouse from May to October 2016. In full anthesis, leaflets were collected, mixed, portioned into 0.5 and 1 g portions, frozen in liquid nitrogen and stored until use at − 80 °C.

### Preparation of potato leaf extract

PLE was prepared as previously described by [16]. Briefly, 0.5 g of frozen leaves was ground in liquid nitrogen, supplemented with 50 ml of distilled water (1% w/v) and shaken for 24 h on a laboratory shaker. Freshly prepared and filtered extract was used for biochemical and mass spectrometry (MS) analysis.

### Evaluation of total glycoalkaloid, total phenolic and total flavonoid contents in potato leaf extract

The TGA concentration was measured for all 166 individuals in 2016 using the colorimetric method by [44] with modification as described by [16]. Briefly, 1% PLE was concentrated fourfold in a vacuum rotary evaporator (SpeedVac Appligene Refrigerated Aspirator, Germany). TGA was extracted using 10% acetic acid and precipitated with 5 M ammonium hydroxide. Samples were suspended in 100% methanol. The colour reaction was carried out using 98% sulfuric acid and 1% paraformaldehyde.

Absorbance was measured on a Hitachi U-1900 (Japan) spectrophotometer at a wavelength of 562 nm against a blank sample. The concentration of TGA was expressed in equivalents of α-solanine (Sigma-Aldrich, S3757).

TP content was determined as in [16] with a modification of the method described by [45]. Analysis was performed on twofold-concentrated PLE using freshly prepared tenfold-diluted Folin–Ciocalteu reagent (Sigma, F9252) and 7.5% sodium carbonate (w/v), and the absorbance was measured at 760 nm against a blank sample. The concentration of TP was expressed as equivalents of gallic acid (PhytoLab, 89,198).

TF was determined as in [16] with a modified method described by [46]. Analysis was performed on twofold-concentrated PLE using 10% aluminium chloride (w/v) and 1 M potassium acetate, and the absorbance was measured at 415 nm against a blank sample. The concentration of TF was expressed as equivalents of quercetin (PhytoLab, No. 89262).

TGA, TP and TF concentrations in selected genotypes were measured in 2016–2018. All measurements were performed in three biological repetitions, and each repetition had two technical replicates.

### Evaluation of phytotoxic potential of potato leaf extract

PP was measured against the test plant - mustard cv. Rota (Vera-Agra Breeding Company, Cieszków, Poland) in three biological repetitions for all 166 individuals in 2016 and for the selected plants in 2017–2018 as previously described by [16]. Briefly, 15 mustard seeds after radicle protrusion (appx. 3 mm long) were transferred into Petri dishes (square, 12 cm) filled with filter paper and moisture with distilled water or 1% PLE (PLE-treated plants). After 5 days of incubation, the lengths of the control and PLE-treated mustard seedlings were measured. PP was expressed in % as the degree of seedling length inhibition/stimulation in relation to the length of control plants (grown in water) according to the formula

$$ Inhibition\ \left(\%\right)=\left(1-\frac{\mathrm{Treated}\ \mathrm{seedling}\ \mathrm{length}\ }{\mathrm{Control}\ \mathrm{seedling}\ \mathrm{length}}\right)x\ 100 $$

### Construction of bulk samples

Based on TGA concentration and PP (Additional File 6), bulk samples C and D were constructed, each with three biological replications. Bulked sample analysis allows for more effective identification of genes underlying a trait. In this approach, contrasting individuals from a segregating population are pooled and then commonly screened to identify specific markers [47]. Sample C exhibited low TGA content (2.7 μg ml^− 1^ in PLE) and high PP (40%); D exhibited a low TGA concentration (2.6 μg ml^− 1^ in PLE) and nondetectable PP (PLE-treated plants were the same length as the control). In each sample, equal amounts of frozen leaves in liquid nitrogen from three F1 individuals were ground, mixed together and stored at − 80 °C.

### RNA isolation and RNA-seq analysis

RNA was isolated from samples C and D according to the protocol described in [48] using TRIzol reagent. Briefly, 0.1 g of tissue ground in liquid nitrogen was supplemented with 1 ml of TRIzol reagent. Extraction was performed twice in chloroform. The RNA was precipitated in 0.3 ml of salt solution (0.8 M sodium citrate and 1.2 M sodium chloride) and 0.3 ml of isopropanol and resuspended in sterile water. The quality and quantity of RNA were determined using a NanoDrop spectrophotometer (Thermo Scientific) at 260 nm and 280 nm and on a 2% agarose gel. Next, RNA was treated with DNase I (Thermo Scientific, EN0521) to degrade double-stranded and single-stranded DNA contaminants in RNA samples.

The mRNA was isolated using the Dynabeads® mRNA Purification Kit for mRNA enrichment (Ambion, 61,006), and a library was prepared using the MGIEasy RNA Directional Library Prep Set (MGI, 1000006386), both according to the manufacturer’s protocols.

The established cDNA libraries were sequenced on the BGISEQ-500 sequencing platform (BGI Genomics, China) to generate 100-bp paired-end reads. RNA-seq reads were generated by Genomed® (Warsaw, Poland). After filtering of adaptor sequences and low-quality reads, data were obtained for subsequent analysis. Then, the index of the reference genome (https://www.ncbi.nlm.nih.gov/assembly/GCF_000226075.1) was built using Bowtie v2.1.0, and clean reads obtained for samples C and D were aligned to the reference genome using TopHat v2.0.9 (Broad Institute, Boston, MA). Next, HTSeq v0.5.3 was used to count the number of reads mapped to each gene. The DEGs were identified by the DESeq package.

### Analysis of gene ontology term enrichment

To study the biological functions of the DEGs, gene set enrichment with GO terms was performed using the topGO package. To extract the significant GO categories, Fisher’s exact test was performed with the *elim* algorithm. To prepare circle diagram of all significant GO terms, we used as query for finding the ontology in various functional categories on the basis of GOslim categories as:

$$ \frac{\mathrm{annotations}\ \mathrm{to}\ \mathrm{terms}\ \mathrm{in}\ \mathrm{GOslim}\ \mathrm{category}\ }{\mathrm{total}\ \mathrm{annotations}\ \mathrm{to}\ \mathrm{terms}\ \mathrm{in}\ \mathrm{this}\ \mathrm{ontology}} \times 100. $$

### Analysis of the glycoalkaloids profile in potato leaf extract

The GA fraction was isolated from 1% PLE of bulked samples C and D using a solid-phase extraction method (QuEChERS). First, for each PLE sample, α-solamarine (ChemFaces, CFN93102) dissolved in methanol was added as an internal standard to a final concentration of 10 ng μl^− 1^ to calculate the percentage of recovery of GA. In the control sample, α-solamarine was added to distilled water to the same final concentration. Both types of samples (control and PLE) were passed through sterilizing filters (0.2 μm, Nalgene™). To 750 μl of a sample, an equal amount of acetonitrile (ACN) with 1% formic acid was added, and the sample was applied onto the solid phase of QuEChERS (UTC, ECQUCHL12CT) and shaken for 30 s on a vortex mixer. Then, the supernatant obtained after GA isolation was diluted 10-fold with methanol. HPLC-MS analysis was performed on a Dionex 3000 RS-HPLC equipped with a DGP-3600 pump, a WPS-3000 TLS TRS autosampler, a TCC-3000 RS column compartment (Dionex Corporation, USA) and a Bruker micrOTOF-QII mass spectrometer (Bruker Daltonics, Germany). The chromatography column was a 50 × 3.1 (i.d)-millimetre Thermo Scientific Hyperil GOLD with 1,9-μm particles (Part No. 25002–052130, Serial No. 0110796A6, Lot No. 10922).

Chromatographic conditions: For the mobile phase, solvent A was water, and solvent B was ACN. The flow program was as follows: 0 min – 5% solvent B; 1.4 min – 5% solvent B; 22.9 min – 95% solvent B; 24.4 min – 95% solvent B; 24.5 min – 5% solvent B; 29 min – 5% solvent B. The injection sample volume was 1.5 μL. The flow rate was 0.2 ml min^− 1^, and the eluent was monitored by MS. The analysis was performed in negative ESI mode. Scan range: 50–1500 m/z, end plate offset: − 500 V, capillary: 4500 V, nebulizer gas (N2): 1,2 bar, dry gas (N2): 10 L/min, dry temperature: 220 °C.

Qualitative analysis of GA content was performed using the frequency of each compound in the TGA found in the sample. The qualitative GA profile was calculated using the following formula:

$$ F\left[\%\right]=\frac{A_n}{A_{A\iota \iota}}\times 100\%, $$

where

*F* – Frequency of compounds

*A*_*n*_ – Area of analysis compound

*A*_*All*_ – Area of all GAs in the sample.

Quantitative analysis was performed using the GA standards α-solanine (ChemFaces, CFN90560), α-chaconine (ChemFaces, CFN00450), α-solamargine (ChemFaces, CFN90159), and α-solasonine (PhytoLab 83,271). To quantify the compounds, calibration curves for each GA standard were generated over the concentration range of 0.1 μg ml^− 1^ to 10 μg ml^− 1^. For leptine II, a curve for α-solanine was used due to the lack of a standard. The results are expressed in μg ml^− 1^ and take into account the percent recovery of each compound in relation to the control sample concentration.

### Protein extraction

Proteins were isolated from C and D as described in [49] with a minor modification. Then, 0.1 g of powdered tissue was suspended in 350 μl of the extraction buffer and incubated on ice for 30 min. Then, phenol solution (Roti®-Aqua-Phenol) was added in a 1:1 (v/v) ratio and incubated at room temperature for 10 min. The phenol phase was recovered twice by centrifugation at 4 °C, transferred to new tubes with extraction buffer 1:1 (v/v) and precipitated in cold methanol containing 0.1 M ammonium acetate 1:4 (v/v). The mixture was incubated overnight at − 20 °C and centrifuged at room temperature. The liquid phase was removed, and the pellet was washed once with 100% methanol pre-chilled to − 20 °C, centrifuged with 80% acetone, and centrifuged at the highest speed. The final protein pellet was air-dried and dissolved in 200 μl of 25 mM ammonium bicarbonate. The sample protein content was determined according to the method described by [50] using the bicinchoninic acid assay and bovine serum albumin as a standard. Five independent biological replicates were analysed in this study. A total of 120 μg of protein from each probe was sent to the Mass Spectrometry Laboratory at the Institute of Biochemistry and Biophysics, Polish Academy of Sciences (Warsaw, Poland), for nano-LC-MS-MS/MS (nanoliquid chromatography coupled to tandem mass spectrometry) analysis.

### Comparative analysis of differentially expressed proteins

Peptide mixtures were analysed by nano-LC-MS-MS/MS using a nano-Acquity (Waters) LC system and a Q-Exactive mass spectrometer (Thermo Electron Corp., San Jose, CA) with the same equipment, buffers and parameters like in [51]. The raw data were processed by Mascot Distiller followed by Mascot Search (Matrix Science, London, UK, on-site licence) against the UniProt *Solanum tuberosum* database (February 2018 release). The search parameters for precursor and product ion mass tolerances were 30 ppm and 0.1 Da, respectively enzyme specificity: trypsin and missed cleavage sites. Peptides with Mascot scores exceeding the threshold value corresponding to < 5% of the expectation value as calculated by the Mascot procedure were considered positively identified. Quantitative analysis was performed as described by [52]. Shortly, the mass calibration and data filtering described above were carried out like in [51]. At the end, lists of identified peptides with corresponding abundances were exported for statistical analysis carried out with in-house developed Diffprot Software (version 1.5.19; 3.01.2013) [53]. Prior to analysis, abundances were normalized with LOWESS. Proteins with more than 90% common peptides were clustered, and only peptides unique for the cluster were used for statistical analysis. Only proteins with q-values ≤0.05 were considered differentially expressed.

## Supplementary Information

**Additional file 1: Supplementary Table S1.** Average retention time (RT) and mass of compounds found in samples D and C.

**Additional file 2: Supplementary Figure S1.** Mass spectra of GAs found in samples C and D.

**Additional file 3: Supplementary Table S2.** All DEGs recognized after comparison D vs. C bulks, sorted by log2 FC.

**Additional file 4: Supplementary Table S3.** GO enrichement of DEGs in molecular function, biological processes and cellular compartments after comparison D vs C.

**Additional file 5: Supplementary Table S4.** Expression pattern of UDP-glucosytransferases and other genes engaged in GAs biosynthesis between the bulks D vs. C, sorted by Log2 FC.

**Additional file 6: Supplementary Table S5.** TGA content and PP in individuals of 15-1 population.

## Data Availability

All data analysed in this study are included in this published article and its supplementary files. All raw and processed RNA-seq data have been deposited in the Gene Expression Omnibus [GEO] repository under the link [https://www.ncbi.nlm.nih.gov/geo/query/acc.cgi?acc=GSE155583] and Sequence Read Archive [SRA] repository under the link [https://www.ncbi.nlm.nih.gov/bioproject/PRJNA650400]. The datasets used and/or analyzed during the current study are available from the corresponding author on reasonable request.

## Abbreviations

CPB: Colorato Potato Beetle
DEGs: differentially expressed genes
DEPs: differentially expressed proteins
GAME: glycoalkaloids metabolism genes
GO: gene ontology
PLE: potato leaf extract
PP: phytotoxic potential
GAs: glycoalkaloids (measured by mass spectrometry)
TF: total flavonoids
TGA: total glycoalkaloids (measured colorimetrically)
TP: total phenolics
