# Correction to: Transcriptional and proteomic insights into phytotoxic activity of interspecific potato hybrids with low glycoalkaloid contents

**DOI:** 10.1186/s12870-022-03574-0

**Published:** 2022-04-11

**Authors:** Katarzyna Szajko, Jarosław Ciekot, Iwona Wasilewicz-Flis, Waldemar Marczewski, Dorota Sołtys-Kalina

**Affiliations:** 1 Plant Breeding and Acclimatization Institute, Młochów Research Centre, Platanowa 19 st, 05-831 Młochów, Poland; 2 Ludwik Hirszfeld Institute of Immunology and Experimental Therapy, Laboratory of Biomedical Chemistry, Rudolfa Weigla 12 st, 53-114 Wrocław, Poland

**Correction to: BMC Plant Biol 21, 60 (2021)**

**https://doi.org/10.1186/s12870-021-02825-w**

Following publication of the original article [1], authors would like to correct the Acknowledgement section. The correct Acknowledgement is given below:

**Acknowledgement**

The equipment used in the Mass Spectrometry Laboratory (Institute of Biochemistry and Biophysics, Polish Academy of Sciences) was sponsored in part by the Centre for Preclinical Research and Technology (CePT), a project co-sponsored by European Regional Development Fund and Innovative Economy, The National Cohesion Strategy of Poland.

We are grateful to Agata Malinowska and Bianka Świderska from Institute of Biochemistry and Biophysics, Mass Spectrometry Laboratory, Polish Academy of Sciences, for the preparation of Figure 1.

The original article has been corrected.
