# Supplementary material for: Transcriptional and proteomic insights into phytotoxic activity of interspecific potato hybrids with low glycoalkaloid contents
Source: BMC Plant Biol. 2021 Jan 22;21:60. doi: 10.1186/s12870-021-02825-w (PMC7825178; doi:10.1186/s12870-021-02825-w)
Supplement: Supplementary file 1 — Additional file 1: Supplementary Table S1. Average retention time (RT) and mass of compounds found in samples D and C. [file 12870_2021_2825_MOESM1_ESM.docx]

**Transcriptional and proteomic insights into phytotoxic activity of interspecific potato hybrids with low glycoalkaloid contents.**

Katarzyna Szajko^1^, Jarosław Ciekot^2^, Iwona Wasilewicz-Flis^1^, Waldemar Marczewski^1^, Dorota Sołtys-Kalina^1^*

^1^ Plant Breeding and Acclimatization Institute, Młochów Research Centre, Platanowa 19 st. 05-831 Młochów, Poland

^2^Ludwik Hirszfeld Institute of Immunology and Experimental Therapy, Laboratory of Biomedical Chemistry, Rudolfa Weigla 12 st., 53-114 Wrocław

Corresponding Author: d.soltys@ihar.edu.pl

Supplementary Table S1. Average retention time (RT) and mass of compounds found in samples D and C

| Number of  compound | Name of compound | Average RT [min] | Average masa [M-H]- |
| --- | --- | --- | --- |
|  | Bulk C |  |  |
| 1 | α-Solasonine | 11.86 | 882.489 |
| 2 | α-Solamargine | 11.94 | 866.493 |
| 3 | α-Solanine | 12.33 | 866.504 |
| 4 | α-Chaconine | 12.34 | 850.499 |
| 5 | Leptine II | 12.94 | 924.500 |
| Bulk D | | | |
| 1 | α-Solasonine | nd* | nd |
| 2 | α-Solamargine | 11.99 | 866.497 |
| 3 | α-Solanine | 12.4 | 866.500 |
| 4 | α-Chaconine | 12.42 | 850.498 |
| 5 | Leptine II | 13.01 | 924.498 |

nd – not detected
