# Supplementary material for: Transcriptional and proteomic insights into phytotoxic activity of interspecific potato hybrids with low glycoalkaloid contents
Source: BMC Plant Biol. 2021 Jan 22;21:60. doi: 10.1186/s12870-021-02825-w (PMC7825178; doi:10.1186/s12870-021-02825-w)

Transcriptional and proteomic insights into phytotoxic activity of interspecific potato hybrids with low glycoalkaloid contents.

Katarzyna Szajko[1], Jarosław Ciekot[2], Iwona Wasilewicz-Flis[1], Waldemar Marczewski[1], Dorota Sołtys-Kalina[1]*

[1] Plant Breeding and Acclimatization Institute, Młochów Research Centre, Platanowa 19 st. 05-831 Młochów, Poland

[2] Ludwik Hirszfeld Institute of Immunology and Experimental Therapy, Laboratory of Biomedical Chemistry, Rudolfa Weigla 12 st., 53-114 Wrocław

Corresponding Author: d.soltys@ihar.edu.pl

Supplementary Figure S1. Mass spectra of GAs found in samples C and D

Bulk C alpha-solasonine

Display Report

Analysis Info

Analysis Name	Z:\Backup\Ekstrakty\C I_GA7_01_5950.d	Acquisition Date	2019-09-28 16:26:07
Method	Metoda neg bez UV short.m	Operator	JC
Sample Name	C I	Instrument	micrOTOF-Q II 10329
Comment			

Acquisition Parameter

Source Type	ESI	Ion Polarity	Negative	Set Nebulizer	1.0 Bar
Focus	Not active	Set Capillary	4500 V	Set Dry Heater	220 °C
Scan Begin	50 m/z	Set End Plate Offset	-500 V	Set Dry Gas	10.0 l/min
Scan End	1500 m/z	Set Collision Cell RF	150.0 Vpp	Set Divert Valve	Source

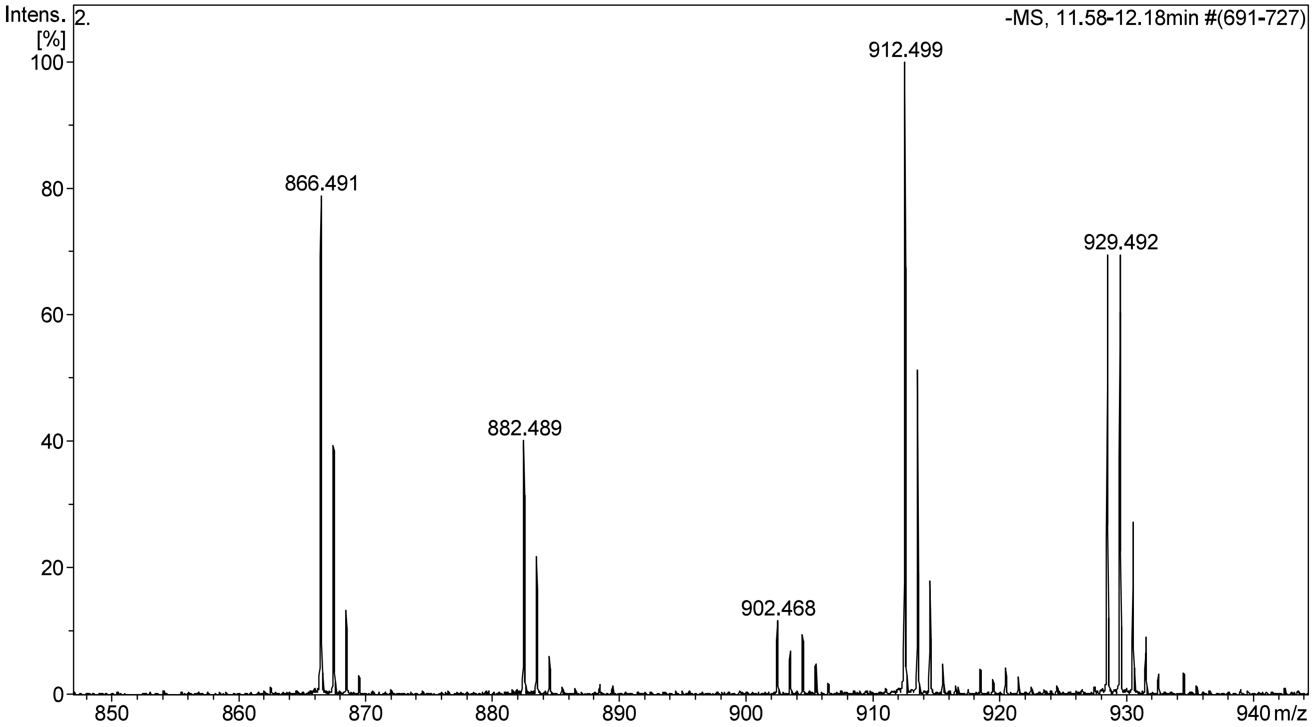

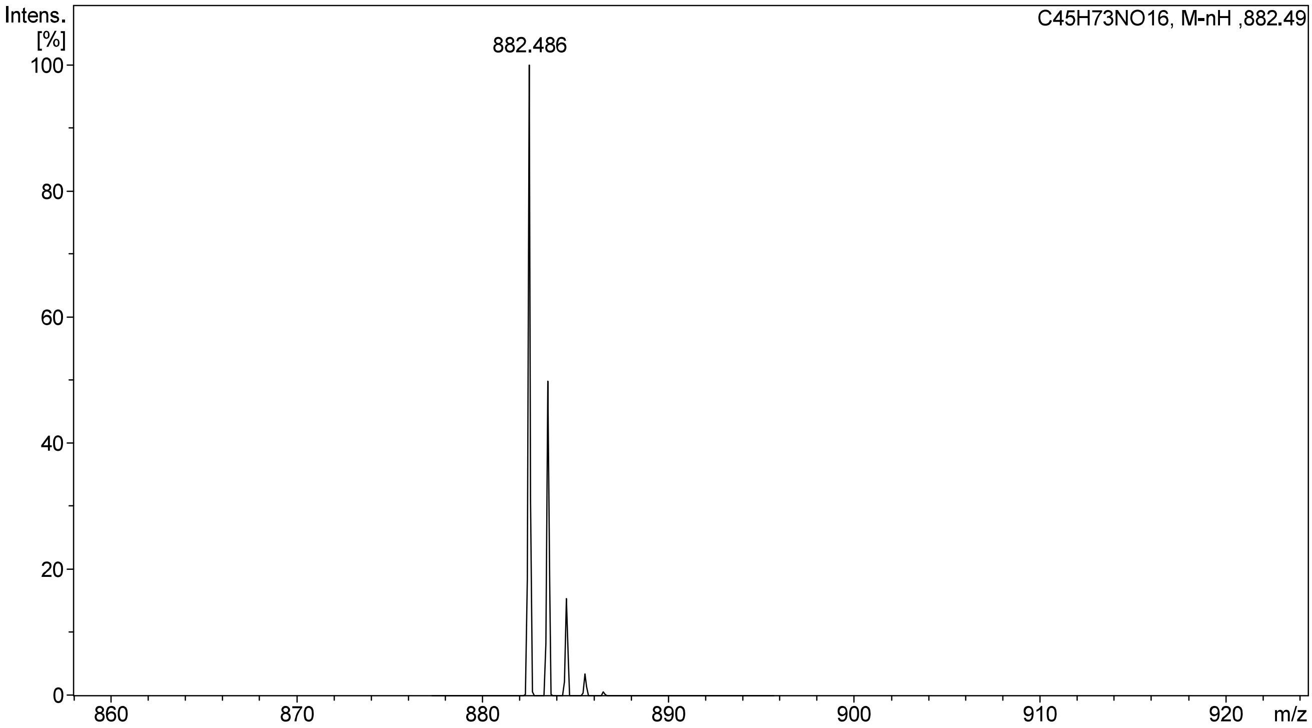

Bulk C alpha-Solamargine

Display Report

Analysis Info

Analysis Name	Z:\Backup\Ekstrakty\C I_GA7_01_5950.d	Acquisition Date	2019-09-28 16:26:07
Method	Metoda neg bez UV short.m	Operator	JC
Sample Name	C I	Instrument	micrOTOF-Q II 10329
Comment			

Acquisition Parameter

Source Type	ESI	Ion Polarity	Negative	Set Nebulizer	1.0 Bar
Focus	Not active	Set Capillary	4500 V	Set Dry Heater	220 °C
Scan Begin	50 m/z	Set End Plate Offset	-500 V	Set Dry Gas	10.0 l/min
Scan End	1500 m/z	Set Collision Cell RF	150.0 Vpp	Set Divert Valve	Source

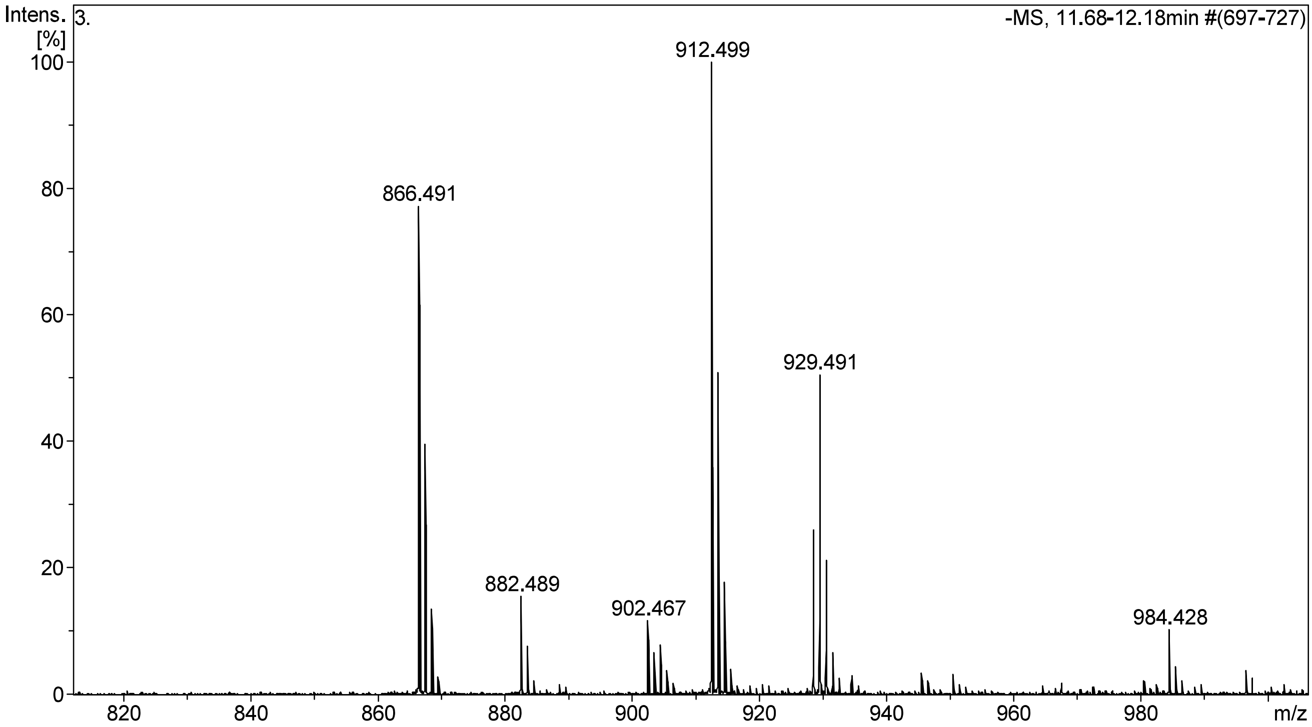

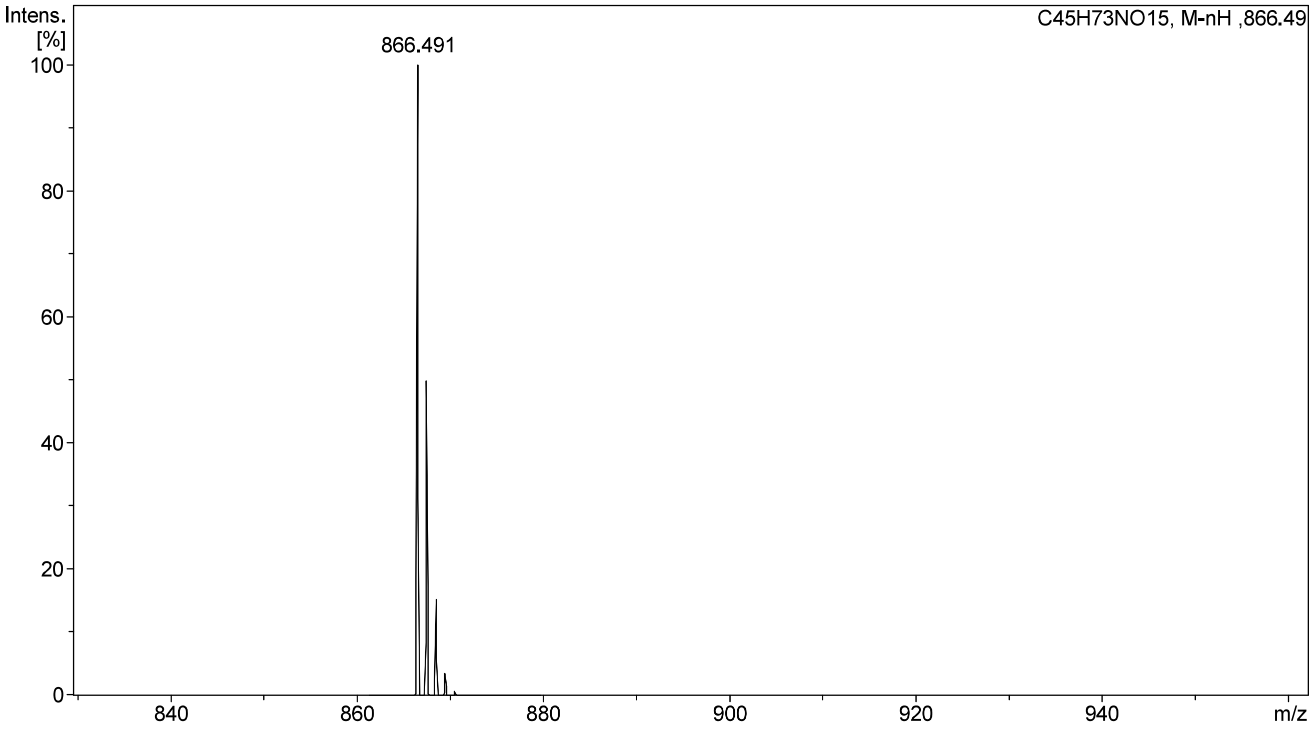

Bulk C alpha-Solanine

Display Report

Analysis Info

Analysis Name	Z:\Backup\Ekstrakty\C I_GA7_01_5950.d	Acquisition Date	2019-09-28 16:26:07
Method	Metoda neg bez UV short.m	Operator	JC
Sample Name	C I	Instrument	micrOTOF-Q II 10329
Comment			

Acquisition Parameter

Source Type	ESI	Ion Polarity	Negative	Set Nebulizer	1.0 Bar
Focus	Not active	Set Capillary	4500 V	Set Dry Heater	220 °C
Scan Begin	50 m/z	Set End Plate Offset	-500 V	Set Dry Gas	10.0 l/min
Scan End	1500 m/z	Set Collision Cell RF	150.0 Vpp	Set Divert Valve	Source

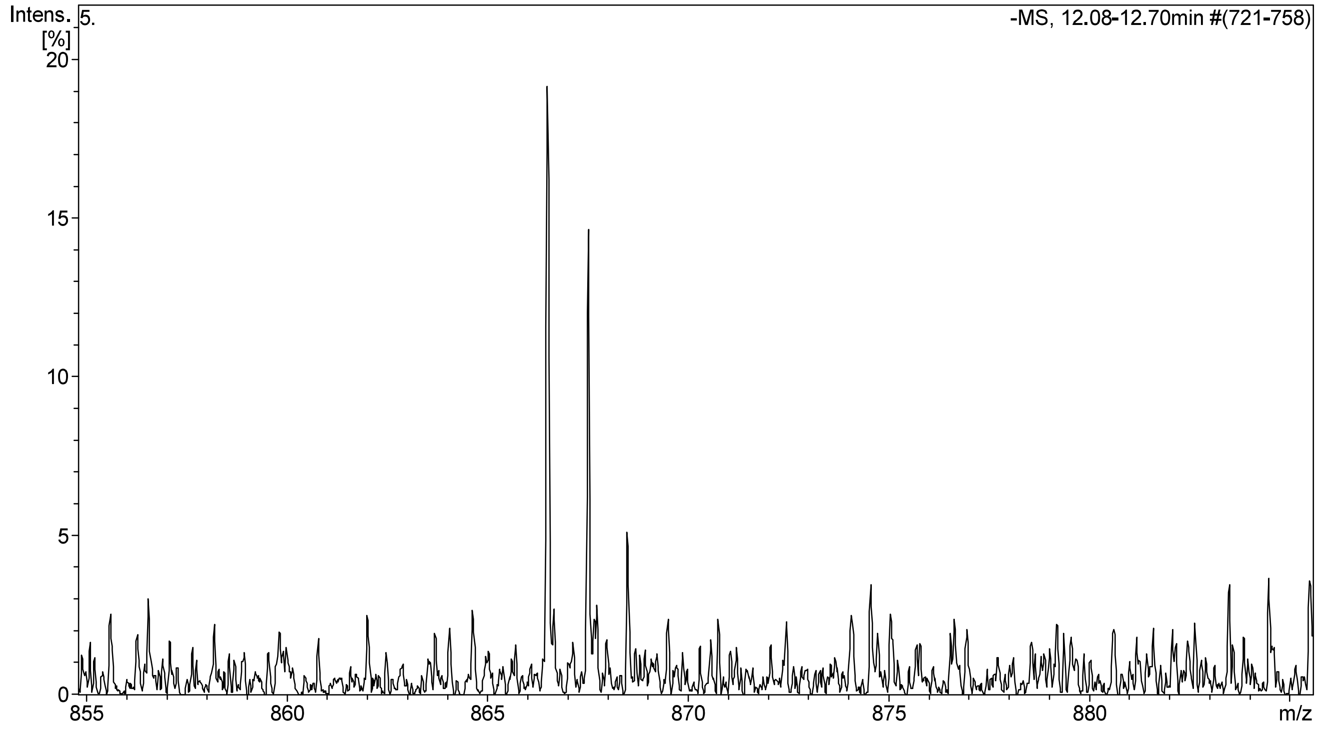

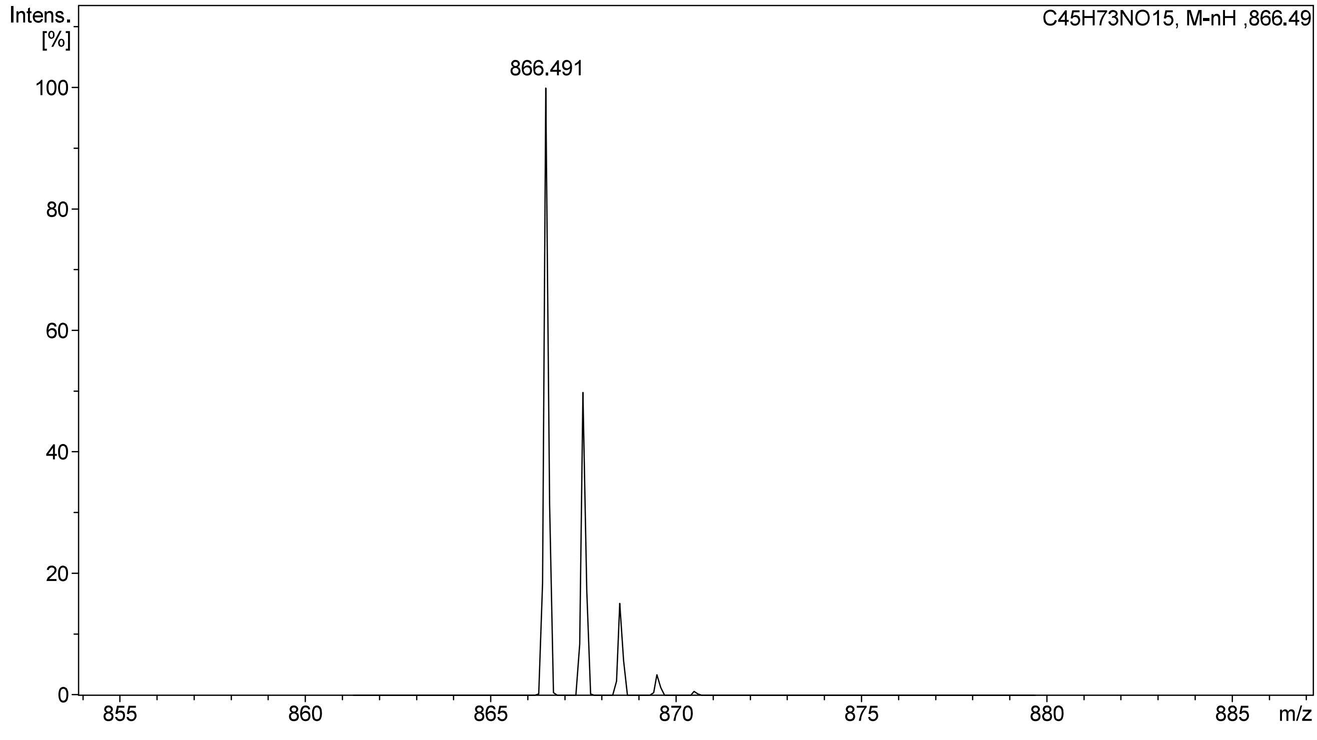

Bulk C alpha-Chaconine

Display Report

Analysis Info

Analysis Name	Z:\Backup\Ekstrakty\C I_GA7_01_5950.d	Acquisition Date	2019-09-28 16:26:07
Method	Metoda neg bez UV short.m	Operator	JC
Sample Name	C I	Instrument	micrOTOF-Q II 10329
Comment			

Acquisition Parameter

Source Type	ESI	Ion Polarity	Negative	Set Nebulizer	1.0 Bar
Focus	Not active	Set Capillary	4500 V	Set Dry Heater	220 °C
Scan Begin	50 m/z	Set End Plate Offset	-500 V	Set Dry Gas	10.0 l/min
Scan End	1500 m/z	Set Collision Cell RF	150.0 Vpp	Set Divert Valve	Source

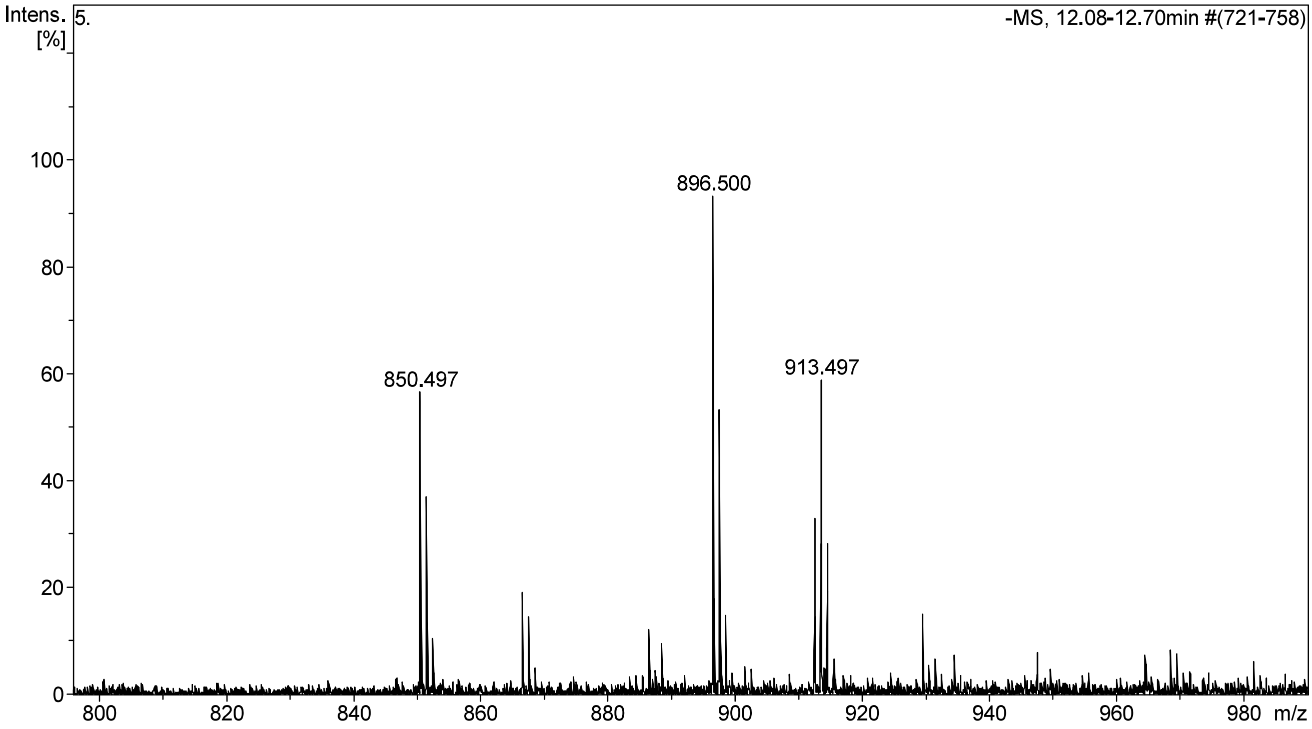

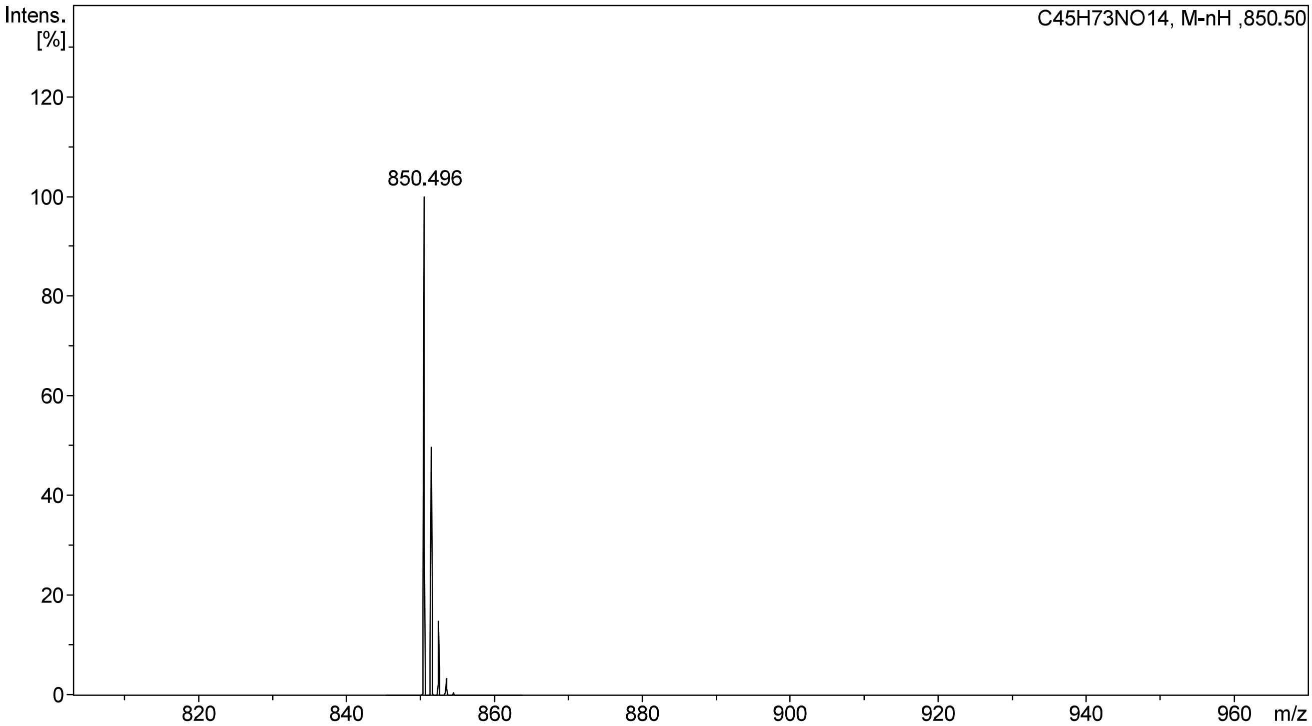

Bulk D alpha-Solamargine

Display Report

Analysis Info

Analysis Name	Z:\Backup\Ekstrakty\D I_GC6_01_5965.d	Acquisition Date	2019-09-29 00:25:32
Method	Metoda neg bez UV short.m	Operator	JC
Sample Name	D I	Instrument	micrOTOF-Q II 10329
Comment			

Acquisition Parameter

Source Type	ESI	Ion Polarity	Negative	Set Nebulizer	1.0 Bar
Focus	Not active	Set Capillary	4500 V	Set Dry Heater	220 °C
Scan Begin	50 m/z	Set End Plate Offset	-500 V	Set Dry Gas	10.0 l/min
Scan End	1500 m/z	Set Collision Cell RF	150.0 Vpp	Set Divert Valve	Source

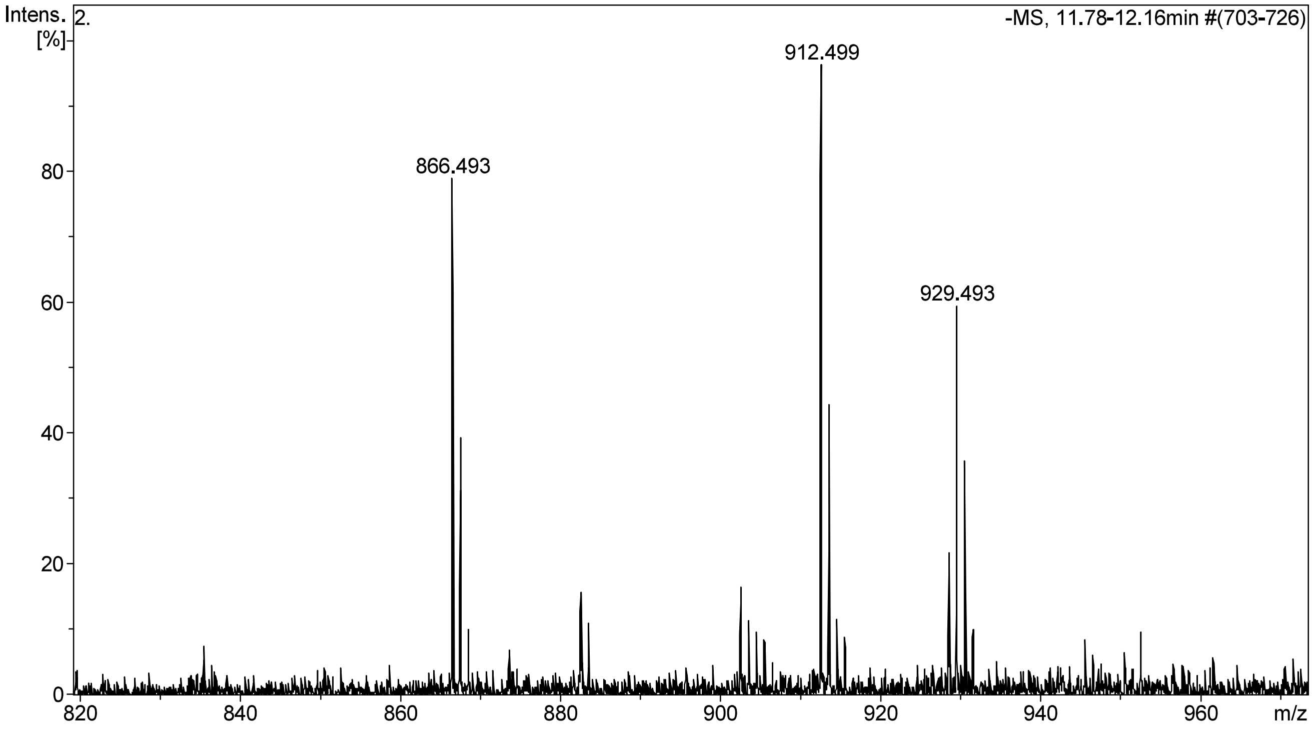

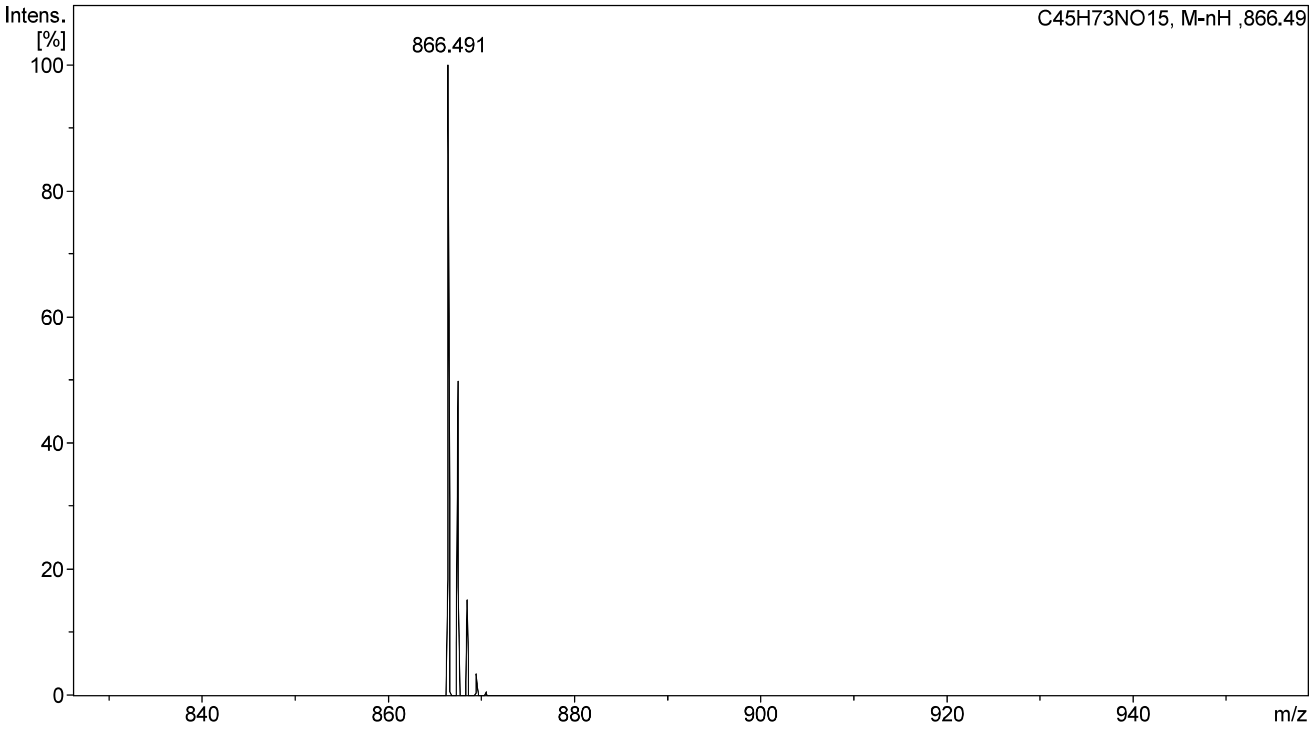

Bulk D alpha-Solanine

Display Report

Analysis Info

Analysis Name	Z:\Backup\Ekstrakty\D I_GC6_01_5965.d	Acquisition Date	2019-09-29 00:25:32
Method	Metoda neg bez UV short.m	Operator	JC
Sample Name	D I	Instrument	micrOTOF-Q II 10329
Comment			

Acquisition Parameter

Source Type	ESI	Ion Polarity	Negative	Set Nebulizer	1.0 Bar
Focus	Not active	Set Capillary	4500 V	Set Dry Heater	220 °C
Scan Begin	50 m/z	Set End Plate Offset	-500 V	Set Dry Gas	10.0 l/min
Scan End	1500 m/z	Set Collision Cell RF	150.0 Vpp	Set Divert Valve	Source

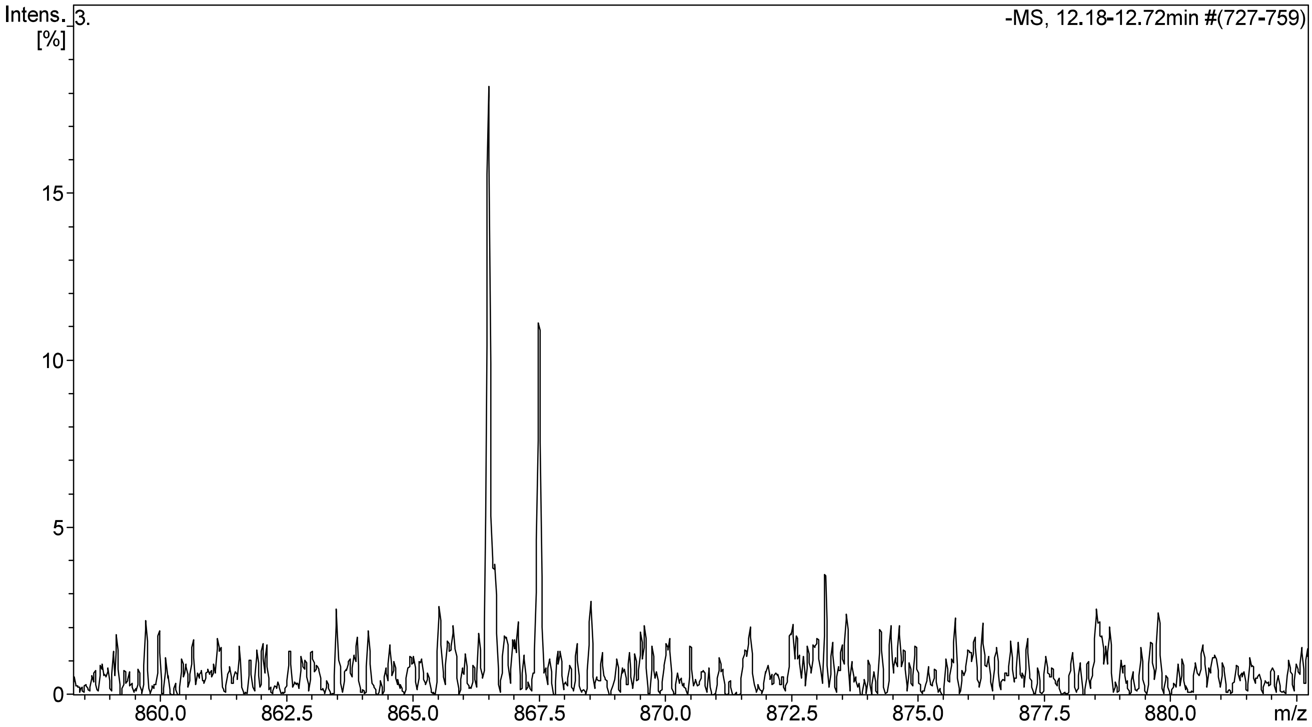

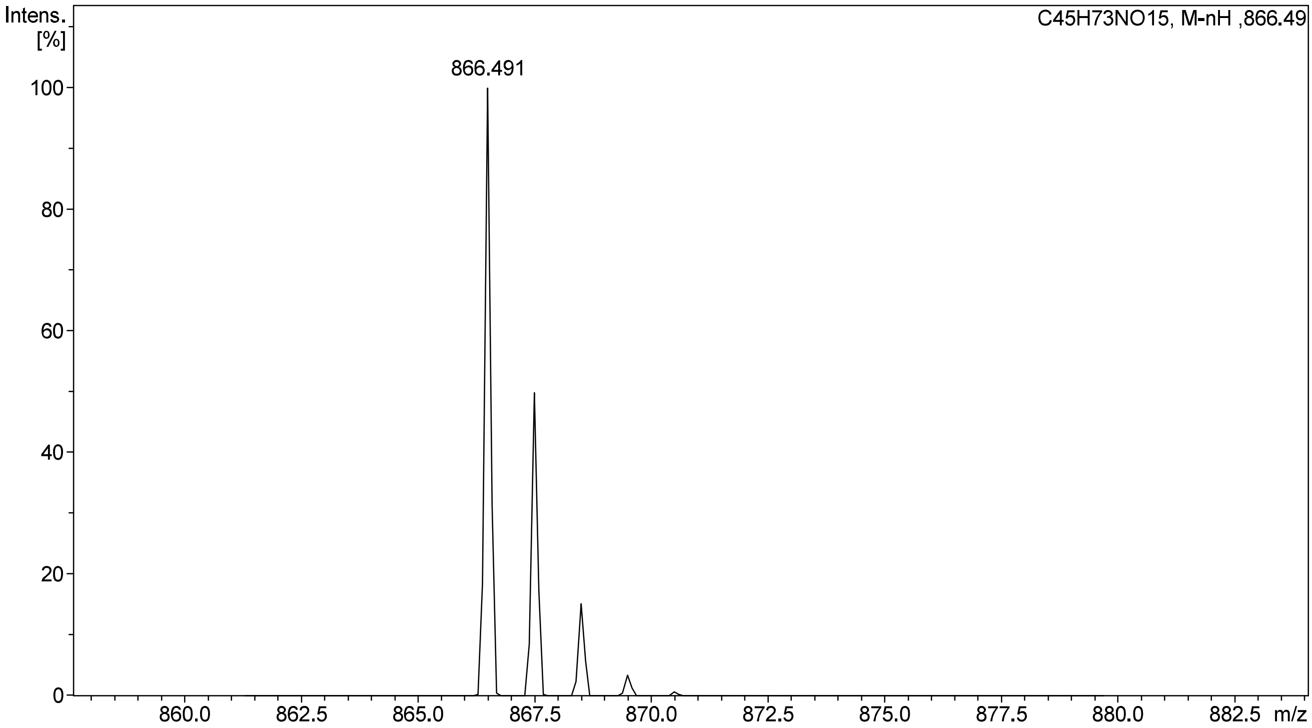

Bulk D alpha-Chaconine

Display Report

Analysis Info

Analysis Name	Z:\Backup\Ekstrakty\D I_GC6_01_5965.d	Acquisition Date	2019-09-29 00:25:32
Method	Metoda neg bez UV short.m	Operator	JC
Sample Name	D I	Instrument	micrOTOF-Q II 10329
Comment			

Acquisition Parameter

Source Type	ESI	Ion Polarity	Negative	Set Nebulizer	1.0 Bar
Focus	Not active	Set Capillary	4500 V	Set Dry Heater	220 °C
Scan Begin	50 m/z	Set End Plate Offset	-500 V	Set Dry Gas	10.0 l/min
Scan End	1500 m/z	Set Collision Cell RF	150.0 Vpp	Set Divert Valve	Source

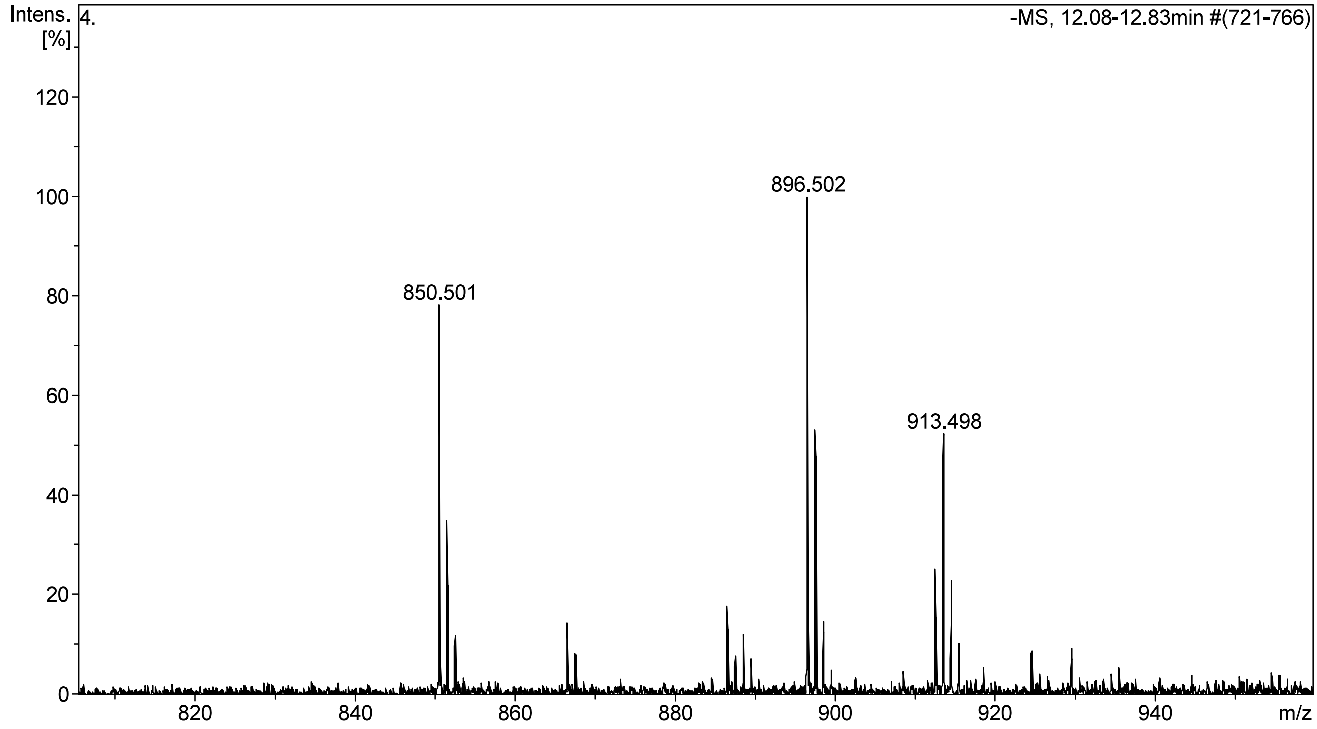

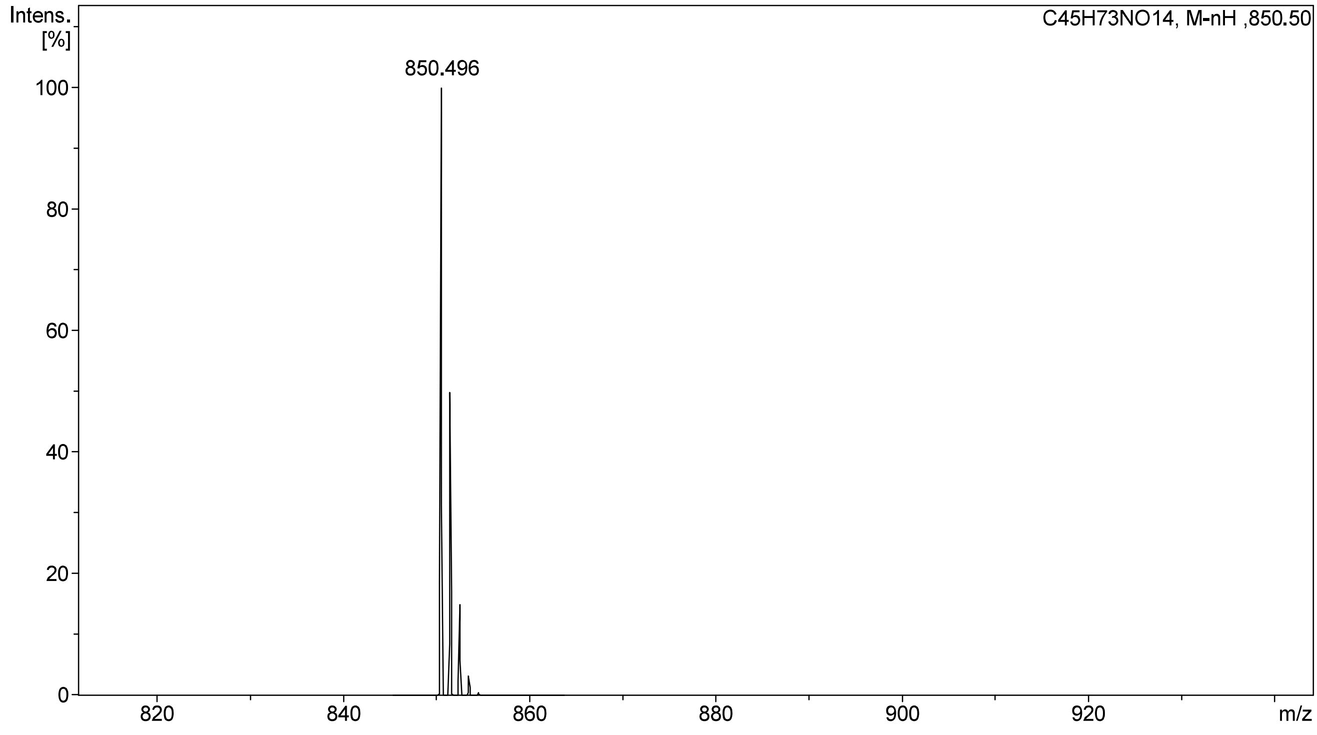

Bulk D Leptine II

Display Report

Analysis Info

Analysis Name	Z:\Backup\Ekstrakty\D I_GC6_01_5965.d	Acquisition Date	2019-09-29 00:25:32
Method	Metoda neg bez UV short.m	Operator	JC
Sample Name	D I	Instrument	micrOTOF-Q II 10329
Comment			

Acquisition Parameter

Source Type	ESI	Ion Polarity	Negative	Set Nebulizer	1.0 Bar
Focus	Not active	Set Capillary	4500 V	Set Dry Heater	220 °C
Scan Begin	50 m/z	Set End Plate Offset	-500 V	Set Dry Gas	10.0 l/min
Scan End	1500 m/z	Set Collision Cell RF	150.0 Vpp	Set Divert Valve	Source

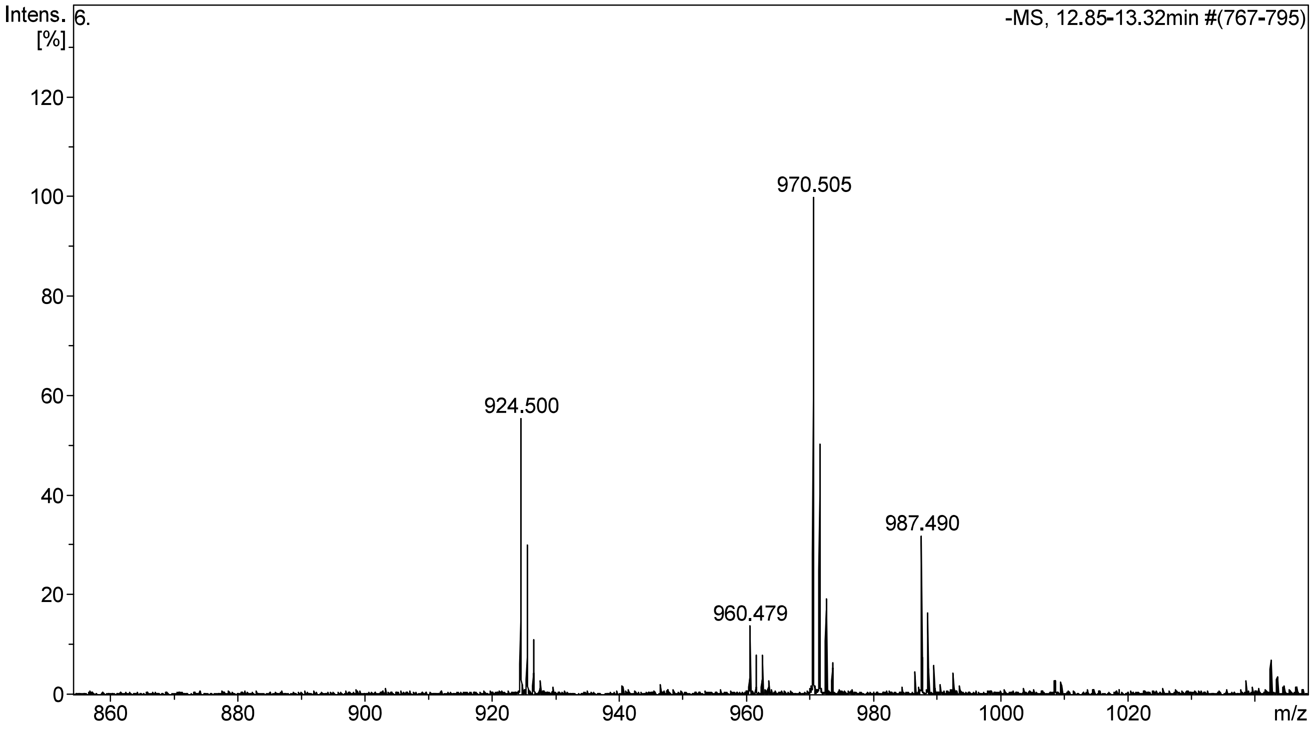

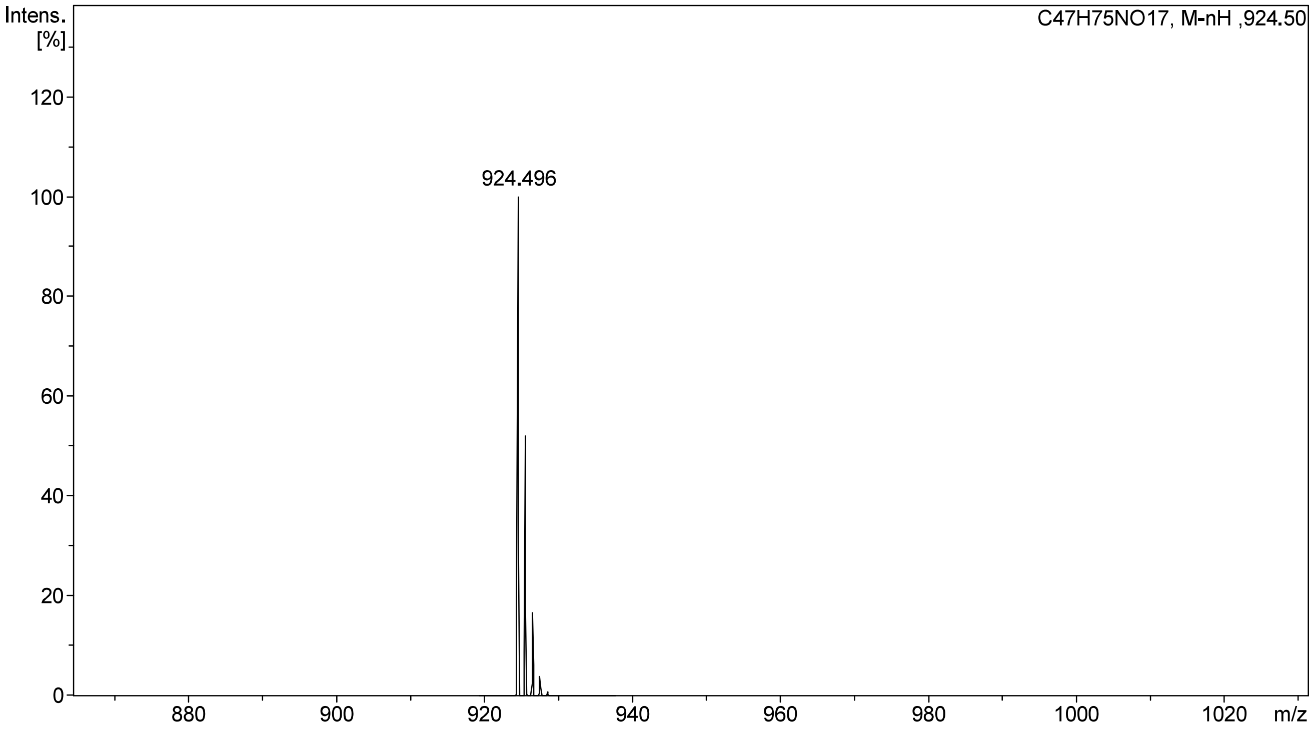

Supplement: Supplementary file 2 — Additional file 2: Supplementary Figure S1. Mass spectra of GAs found in samples C and D. [file 12870_2021_2825_MOESM2_ESM.pdf]
