# Supplementary material for: Transcriptional and proteomic insights into phytotoxic activity of interspecific potato hybrids with low glycoalkaloid contents
Source: BMC Plant Biol. 2021 Jan 22;21:60. doi: 10.1186/s12870-021-02825-w (PMC7825178; doi:10.1186/s12870-021-02825-w)
Supplement: Supplementary file 5 — Additional file 5: Supplementary Table S4. Expression pattern of UDP-glucosytransferases and other genes engaged in GAs biosynthesis between the bulks D vs. C, sorted by Log2 FC. [file 12870_2021_2825_MOESM5_ESM.docx]

Supplementary Table S4. Expression pattern of UDP-glucosytransferases and other genes* engaged in GAs biosynthesis between the bulks D vs. C, sorted by Log2 FC

| **Locus** | **log2 FC** | **FDR** | | **Product** | |  |
| --- | --- | --- | --- | --- | --- | --- |
| LOC102584658 | -3.01 | | 1.21454E-06 | | UDP-glycosyltransferase 90A1-like | |
| LOC102579078 | -1.37 | | 8.22628E-16 | | UDP-glycosyltransferase 89B2-like | |
| LOC107059319 | -1.22 | | 3.66624E-06 | | UDP-glycosyltransferase 86A1-like | |
| LOC107059441 | -1.21 | | 0.0008462 | | UDP-glycosyltransferase 86A1-like | |
| LOC102578982 | -1.19 | | 3.27837E-17 | | UDP-glycosyltransferase 86A1-like | |
| LOC102590649 | -0.98 | | 1.45689E-06 | | UDP-glycosyltransferase 92A1-like | |
| LOC102600509 | -0.96 | | 2.0451E-11 | | UDP-glycosyltransferase 86A1-like | |
| LOC102599178 | -0.95 | | 3.76124E-08 | | UDP-glycosyltransferase 91A1-like | |
| LOC102592659 | -0.87 | | 0.000343603 | | UDP-glycosyltransferase 90A1-like | |
| LOC102592932 | -0.86 | | 0.001432503 | | UDP-glycosyltransferase 73C1-like | |
| LOC102592777 | -0.83 | | 0.000605113 | | UDP-glycosyltransferase 86A1-like | |
| LOC102582377 | -0.82 | | 0.007069654 | | UDP-glycosyltransferase 73C3-like | |
| LOC107062557 | -0.74 | | 2.06596E-12 | | UDP-glycosyltransferase 90A1-like. transcript variant X1 | |
| LOC102606101 | -0.66 | | 8.12624E-07 | | UDP-glycosyltransferase 74F2-like | |
| LOC102594178 | -0.63 | | 0.000184432 | | UDP-glycosyltransferase 74E2-like | |
| LOC102579484 | -0.63 | | 3.06488E-07 | | protein SGT1 homolog A-like. transcript variant X2 | |
| LOC102583510 | -0.43 | | 0.047676366 | | UDP-glycosyltransferase 87A2-like | |
| LOC102593972 | -0.36 | | 0.014300153 | | UDP-glycosyltransferase 73C3-like | |
| LOC102600382 | 0.43 | | 0.031165619 | | UDP-glycosyltransferase 79B6-like | |
| LOC102579214 | 0.48 | | 0.005470566 | | UDP-glycosyltransferase 89A2-like | |
| LOC102583835 | 0.63 | | 2.57156E-07 | | UDP-glycosyltransferase 87A2-like | |
| LOC102577928 | 0.69 | | 0.040547647 | | rhamnose:beta-solanine/beta-chaconine rhamnosyltransferase | |
| LOC102596452 | 1.05 | | 1.42977E-07 | | UDP-glycosyltransferase 91C1 | |

*selected based on FDR<0.05 from RNA-seq experiment
